# The Intelligent Evolution of Radar Signal Deinterleaving: A Systematic Review from Foundational Algorithms to Cognitive AI Frontiers

**DOI:** 10.3390/s26010248

**Published:** 2025-12-31

**Authors:** Zhijie Qu, Jinquan Zhang, Yuewei Zhou, Lina Ni

**Affiliations:** 1 College of Computer Science and Engineering, Shandong University of Science and Technology, Qingdao 266590, China; zhijiequ@sdust.edu.cn (Z.Q.);; 2 Key Laboratory of the Ministry of Education for Embedded System and Service Computing, Shanghai 201804, China

**Keywords:** radar signal deinterleaving, electronic countermeasures, pulse sorting, deep learning, clustering algorithms

## Abstract

The escalating complexity, density, and agility of the modern electromagnetic environment (CME) pose unprecedented challenges to radar signal deinterleaving, a cornerstone of electronic intelligence. While traditional methods face significant performance bottlenecks, the advent of artificial intelligence, particularly deep learning, has catalyzed a paradigm shift. This review provides a systematic, comprehensive, and forward-looking analysis of the radar signal deinterleaving landscape, critically bridging foundational techniques with the cognitive frontiers. Previous reviews often focused on specific technical branches or predated the deep learning revolution. In contrast, our work offers a holistic synthesis. It explicitly links the evolution of algorithms to the persistent challenges of the CME. We first establish a unified mathematical framework and systematically evaluate classical approaches, such as PRI-based search and clustering algorithms, elucidating their contributions and inherent limitations. The core of our review then pivots to the deep learning-driven era, meticulously dissecting the application paradigms, innovations, and performance of mainstream architectures, including Recurrent Neural Networks (RNNs), Transformers, Convolutional Neural Networks (CNNs), and Graph Neural Networks (GNNs). Furthermore, we venture into emerging frontiers, exploring the transformative potential of self-supervised learning, meta-learning, multi-station fusion, and the integration of Large Language Models (LLMs) for enhanced semantic reasoning. A critical assessment of the current dataset landscape is also provided, highlighting the crucial need for standardized benchmarks. Finally, this paper culminates in a comprehensive comparative analysis, identifying key open challenges such as open-set recognition, model interpretability, and real-time deployment. We conclude by offering in-depth insights and a roadmap for future research, aimed at steering the field towards end-to-end intelligent and autonomous deinterleaving systems. This review is intended to serve as a definitive reference and insightful guide for researchers, catalyzing future innovation in intelligent radar signal processing.

## 1. Introduction

### 1.1. Background and Significance of Radar Signal Deinterleaving

**Figure 1:**
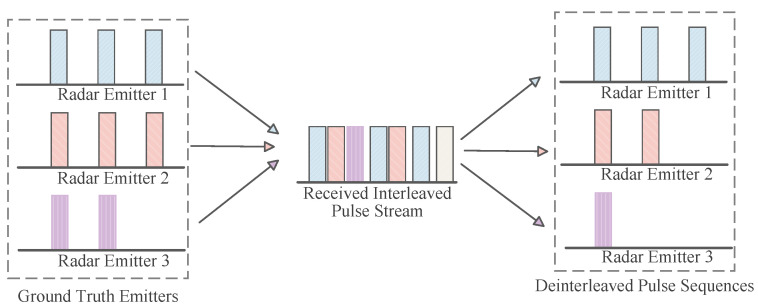
Schematic Diagram of Radar Emitter Deinterleaving.

**Figure 2:**
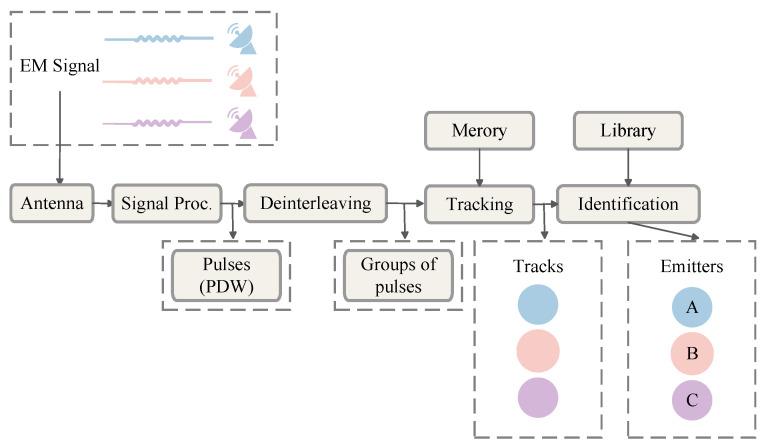
Passive Receiver Chain [4].

In modern Electronic Warfare (EW) and Electronic Intelligence (ELINT) domains, the effective reception, processing, and identification of signals from non-cooperative radar emitters are crucial for gaining battlefield initiative [1,2]. Radar Signal Deinterleaving, also known as pulse train separation, is a critical process in electronic reconnaissance. Upon receiving interleaved pulse streams from multiple emitters, the system separates them into independent sequences. Ultimately, these sequences are associated with specific emitters [3,4,5]. This entire process is schematically depicted in Figure 1. The subsequent signal processing relies entirely on the quality of the data extracted from the front-end hardware. A typical passive receiver chain, which precedes the deinterleaving engine, is illustrated in Figure 2. The accuracy and real-time performance of deinterleaving are pivotal to the overall effectiveness of an Electronic Support Measures (ESM) system, holding profound strategic significance for battlefield situational awareness, threat warning, and the implementation of effective electronic countermeasures [6,7].

### 1.2. Evolution of Radar Signal Deinterleaving Technology

The technological evolution of radar signal deinterleaving chronicles a continuous struggle against the escalating CME. This trajectory is marked by a fundamental paradigm shift from model-driven approaches, which rely on predefined physical models and deterministic rules, to data-driven methodologies that autonomously learn complex latent patterns from vast datasets. However, radar systems are becoming increasingly diverse, and signal environments are exhibiting exponential complexity. Faced with CME challenges such as high density, parameter agility, and weak signals, the performance bottlenecks of traditional methods are becoming increasingly prominent. Furthermore, existing technical reviews often focus on specific technical branches or were published some time ago, failing to comprehensively and systematically integrate the technological transformations brought about by deep learning and emerging paradigms in recent years, and to conduct in-depth critical analyses specifically addressing CME challenges.

This review is precisely situated against this backdrop, offering a highly systematic, critical, and forward-looking comprehensive assessment. Unlike previous reviews that emphasized single technical branches or model overviews, the core unique perspective of this paper lies in closely integrating the intrinsic logic of technological evolution with the external challenges posed by the modern CME. With unprecedented comprehensiveness, it consolidates a technological panorama spanning from classical methods to the frontiers of deep learning, and further to the latest emerging paradigms, thereby constructing a complete picture for readers that traverses three major technological phases and extends towards future development directions. We not only outline the theoretical foundations and evolutionary trajectories of various methods but also crucially reveal their intrinsic connections, the logic of technological leaps, and their advantages, limitations, and driving factors when addressing core CME challenges (such as high density, parameter agility, LPI, and open-set recognition). Through in-depth analysis and critical evaluation, this paper aims to provide a truly guiding reference for promoting continuous innovation and development in intelligent radar signal processing technologies.

#### 1.2.1. The Golden Age of Deterministic Temporal Models

In the nascent stages, characterized by relatively sparse electromagnetic environments and simple radar waveforms, PRI served as a stable and unique “fingerprint” for emitter identification. The prevailing algorithmic philosophy was predicated on the assumption of temporal regularity: identifying stable or simple periodic patterns within the pulse Time of Arrival (TOA) sequence. Radar signal deinterleaving technology has evolved from simple to complex, and from manual rules to intelligent learning. Early methods primarily relied on analyzing the Time of Arrival (TOA) of pulses, performing deinterleaving by searching for stable PRIs. Typical representatives include the Cumulative Difference of Inter-Pulse Intervals (CDIF) histogram and the Sequential Difference of Inter-Pulse Intervals (SDIF) methods [8,9]. These methods visualize the inherent periodic patterns in the time domain by accumulating “votes” in the TOA difference domain, manifesting them as energy peaks in histograms. They performed excellently in scenarios with fixed or simply staggered PRIs, forming the cornerstone of deinterleaving technology. However, their success was built upon a fragile strong assumption: that the PRI pattern is simple and predictable. Once complex PRI modulations or moderate to high levels of pulse loss were encountered, their performance would plummet due to interference from “false peaks” and the masking of true peaks.

#### 1.2.2. Statistical Clustering in Multi-Dimensional Parameter Space

With the widespread adoption of PRI agility by radars to counter reconnaissance and improve their own performance, the path of relying solely on TOA temporal features gradually reached a dead end. Researchers were forced to broaden their horizons, extending their focus from one-dimensional time series to feature spaces composed of multiple parameters such as Direction of Arrival (DOA), Radio Frequency (RF), and Pulse Width (PW). The deinterleaving problem was thus redefined as a classic unsupervised learning problem: data clustering. Subsequently, researchers reframed the problem as data clustering in a multi-dimensional parameter space, utilizing algorithms like K-Means and DBSCAN to jointly cluster multiple pulse parameters (e.g., DOA, RF, PW) [10,11]. The core idea of these methods is “birds of a feather flock together,” implying that pulses from the same emitter should be spatially close in the multi-dimensional parameter space, forming cluster distributions. This transition enhanced the comprehensive utilization of multiple parameters and marked the evolution of deinterleaving technology from temporal analysis to multi-dimensional statistical pattern recognition. However, these “shallow” machine learning methods still faced three inherent bottlenecks: first, heavy reliance on “feature engineering,” where algorithmic performance is significantly influenced by feature selection, weighting, and normalization, requiring extensive expert experience; second, “hyperparameter sensitivity,” such as the K value in K-Means and the neighborhood parameters in DBSCAN, which are difficult to set a priori in non-cooperative scenarios with unknown emitter counts; and third, they are mostly based on simple Euclidean distance or density connectivity assumptions, making it difficult to effectively partition pulse clusters with complex shapes, ambiguous boundaries, and uneven densities in the parameter space.

#### 1.2.3. Deep Learning-Led Data-Driven Revolution

Entering the 21st century, particularly over the past decade, advancements in machine learning, especially deep learning, have provided entirely new solutions for radar signal deinterleaving [12,13,14,15,16,17,18,19,20,21]. Deep neural networks, with their powerful nonlinear mapping and hierarchical feature learning capabilities, signify a fundamental paradigm shift from “model-driven” to “data-driven”. They eliminate the need for complex manually designed rules or feature extractors, enabling them to automatically learn advanced abstract features required for distinguishing different emitters directly from raw or simply preprocessed pulse data. Researchers began to treat pulse sequences as special time series or images, leveraging the powerful nonlinear feature extraction and pattern recognition capabilities of deep neural networks to solve the deinterleaving problem from a “data-driven” perspective. This end-to-end learning paradigm has not only significantly surpassed traditional methods in performance, especially in robustness when handling high-density, highly overlapped, and complex modulated signals, but has also given rise to numerous innovative methodological paradigms, such as segmentation-based, object detection-based, and graph-based deinterleaving models [22,23,24], opening up new and imaginative avenues for solving intelligent deinterleaving problems in previously intractable non-cooperative scenarios.

### 1.3. Challenges Posed by Modern Complex Electromagnetic Environments

With the rapid development of radar technology and the increasing complexity of battlefield frequency usage strategies, modern battlefield electromagnetic environments exhibit unprecedented complexity, posing severe challenges to traditional radar signal deinterleaving [25,26,27]. These challenges are not merely quantitative accumulations but represent qualitative shifts, profoundly exposing the fundamental limitations of traditional deinterleaving methods.

#### 1.3.1. High-Density Overlap and Feature Ambiguity

The number of radar emitters in the battlefield space has increased dramatically, resulting in extremely high pulse densities captured by reconnaissance receivers per unit of time. This first triggers a “combinatorial explosion” at the algorithmic level: for a sequence containing N pulses, the number of potential pulse-emitter association hypotheses grows exponentially, rendering search-based traditional methods computationally infeasible. More critically, pulses from different emitters severely overlap in the temporal, frequency, and spatial domains. This leads to highly ambiguous or even completely fused boundaries of originally distinct “clusters” in the multi-dimensional parameter space, causing the separation difficulty to increase exponentially [28,29]. This severe feature confusion directly challenges all clustering algorithms based on the assumption of feature space separability, making it difficult to find effective separating hyperplanes.

#### 1.3.2. Agile Emitters and Dynamic Feature Invalidation

The widespread application of Multi-Function Radars (MFRs) and Phased Array Radars (PARs) enables radars to rapidly change or jitter their operating modes, waveform patterns, and parameters (e.g., PRI, RF, PW) within a short time frame [30,31,32]. The core characteristic of these radars is the high agility and uncertainty of their operating modes and parameters.

PRIs are no longer stable “fingerprints”. Instead, they exhibit complex modulation forms such as staggering, sliding, grouping, and jittering. They may even employ mixed modes, rendering traditional methods based on stable PRI assumptions ineffective. Concurrently, inter-pulse frequency agility, rapid beam scanning, and switching of intra-pulse modulation methods cause emitters to exhibit multiple seemingly unrelated characteristics within a short period, akin to a “chameleon” adept at camouflage. This places extremely high demands on the dynamic adaptation and pattern capture capabilities of deinterleaving algorithms.

#### 1.3.3. Low SNR/LPI and Input Quality Degradation

To enhance survivability, modern radars generally adopt LPI designs, such as low power and wide bandwidth, and frequency diversity. This strikes a double blow to the deinterleaving task. Firstly, it causes signals to be submerged in noise and interference, increasing the difficulty of detection and precise parameter measurement [33]. Low Signal-to-Noise Ratio (SNR) signals lead to a significant increase in pulse loss rate, severely undermining the completeness of the sequence on which PRI and other temporal features rely. Secondly, low SNR introduces larger parameter measurement errors, causing jitter in otherwise stable parameters (e.g., RF, PW), further exacerbating feature space ambiguity. Deinterleaving algorithms must not only process ideal Pulse Description Words (PDWs) but also exhibit high robustness to such “dirty data” filled with noise, losses, and errors.

#### 1.3.4. Open-Set Recognition of Novel Emitters

Continuous iteration in military technology signifies the emergence of new radar systems, inevitably leading reconnaissance systems to encounter unprecedented signal patterns. Traditional deinterleaving methods and most supervised learning models operate under a “closed-set” assumption, meaning all signal classes to be deinterleaved are known. When unknown signals appear, these models may erroneously “force” them into the most similar known category, leading to catastrophic misjudgments. Therefore, deinterleaving algorithms must possess the capability to discover and handle unknown emitters (Open Set) [34]. This requires algorithms to evolve from simple “classifiers” to “cognitive systems” capable of discovery and adaptation, meaning they should not only accurately separate known signals but also effectively detect “this is a new target” and establish a new class archive for it.

### 1.4. Contributions and Structure of This Paper

**Figure 3:**
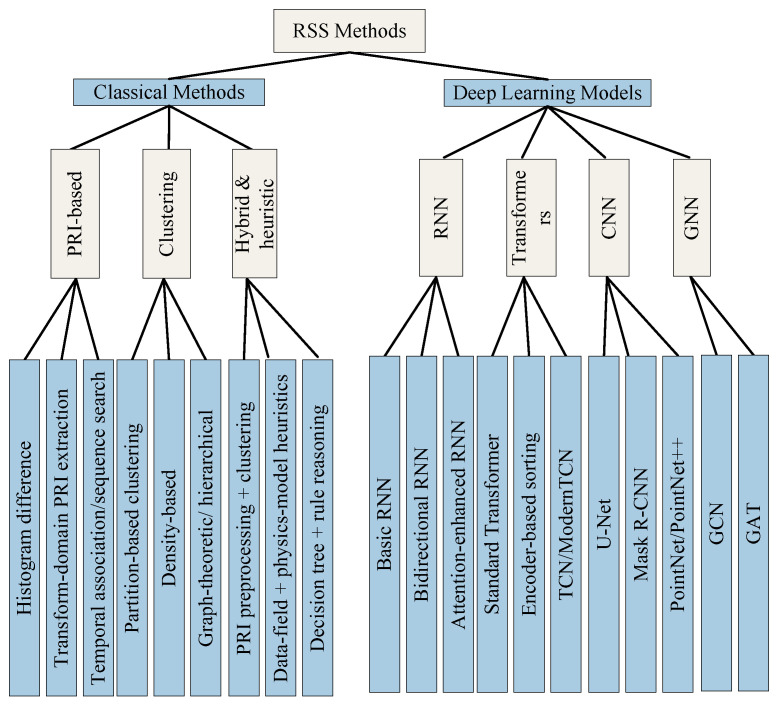
Evolutionary Path of Radar Signal Deinterleaving Technology.

The overall structure of this paper, detailing the progression from foundational concepts to advanced paradigms, is visually summarized by the Radar Signal Deinterleaving Technology Development Roadmap shown in Figure 3. Although related reviews have retrospectively covered radar signal deinterleaving technologies [35,36], existing literature predominantly concentrates on specific technical branches or were published earlier. They fail to comprehensively and systematically integrate the paradigm shifts, primarily driven by deep learning in recent years, along with their latest advancements and emerging development directions in addressing the challenges of the CME. In contrast, this paper aims to provide a highly systematic and forward-looking comprehensive review. Its unique contributions are manifested as follows:

- Comprehensive Perspective on Evolution and Integration: This paper constructs an evolutionary roadmap of radar signal deinterleaving technologies that spans three major technical phases (classical temporal models, statistical clustering, deep learning) and extends to cutting-edge emerging paradigms. We not only delineate the theoretical foundations and technical trajectories of various methods but also focus on revealing their intrinsic interconnections, the logic of technological evolution, and their synergistic and complementary potential in addressing modern CME challenges, thereby offering a unified, integrated technological panorama.
- In-depth Critical Analysis Targeting CME Challenges: Diverging from reviews that merely describe algorithmic flows, this paper centers its analysis on the severe challenges posed by modern complex electromagnetic environments (such as high density, parameter agility, LPI, and open-set recognition). We deeply dissect the core advantages, inherent limitations, and technical bottlenecks of various methods (including classical and deep learning approaches) in confronting these specific challenges. Based on this, we present critical evaluations and syntheses, providing insights into the underlying reasons for the current technological status.
- Focus on Latest Frontiers and Key Driving Factors: This paper keenly tracks and thoroughly analyzes the latest research outcomes from the past three years, with a particular emphasis on representative frontier technologies such as Transformers, GNNs, TCNs, self-supervised learning, meta-learning, and LLMs. We not only enumerate the applications of these technologies in deinterleaving tasks but also profoundly reveal them (e.g., self-attention, graph structure modeling, data-driven representation learning) as key driving factors for tackling CME challenges. Building upon this, we offer insightful predictions and outlooks for future development directions. These directions include enhancing generalization to unknown signals and few-shot learning capabilities; achieving real-time performance via edge computing; ensuring model interpretability and robustness; and developing end-to-end cognitive electronic warfare systems. Crucially, we also explore the transformative potential of large language models to empower semantic cognition and reasoning in this domain.
- Establishing Benchmarks: Critically Assessing Key Datasets: Addressing the critical need for standardized benchmarks in radar signal deinterleaving (RSS) research—a gap often hindering reproducible results and comparative validation—this paper provides a detailed overview and critical assessment of existing datasets. We specifically highlight the characteristics and significance of resources such as the datasets from the the 2nd Electromagnetic Big Data Super Contest (EBDSC).
  By characterizing their strengths, complexity, and suitability for evaluating modern DL techniques, we underscore their role in providing a crucial foundation for reproducible research and robust assessment of deinterleaving algorithms against realistic challenges, thereby helping to establish much-needed benchmarks in the field.
- Structured and Readable Presentation: Through a clearly designed structural framework, comprehensive method comparison tables, and summary tables appended after each major chapter, this paper aids readers in rapidly grasping core content and constructing a clear knowledge system. This structured presentation not only facilitates quick comprehension of key information but, more importantly, explicitly identifies critical open issues and unexplored research gaps in current research, thereby providing a highly focused and exceptionally guiding reference for researchers in related fields.

**Figure 4:**
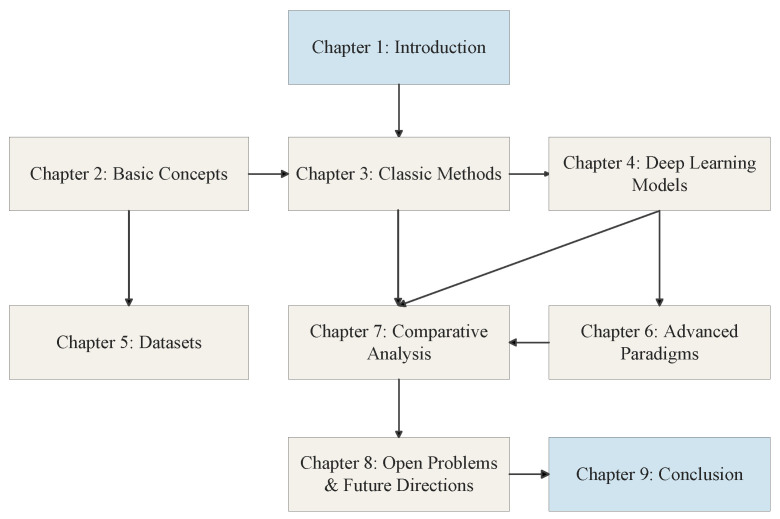
Structural Diagram of This Paper.

As shown in Figure 3, the structure of this paper is organized as follows: Section 2 introduces the basic concepts of radar signal deinterleaving, including problem modeling, key parameters, and performance evaluation metrics. Section 3 reviews and critically analyzes classical deinterleaving methods based on PRI analysis and traditional clustering. Section 4 elaborates on the applications of mainstream deep learning models in radar signal deinterleaving, covering Recurrent Neural Networks, Transformers, Convolutional Neural Networks, and Graph Neural Networks. Section 5 discusses the current status of datasets in the field. Section 6 explores advanced and emerging deinterleaving paradigms such as self-supervised learning, meta-learning, and multi-station fusion. Section 7 provides a comprehensive comparative analysis of all discussed methods. Section 8 summarizes the open issues facing the field and forecasts future research directions. Finally, Section 9 concludes the paper. The logical flow and structural layout of this comprehensive review are visually summarized in Figure 4.

## 2. Fundamentals of Radar Signal Deinterleaving

### 2.1. Signal Model and Problem Definition

**Figure 5:**
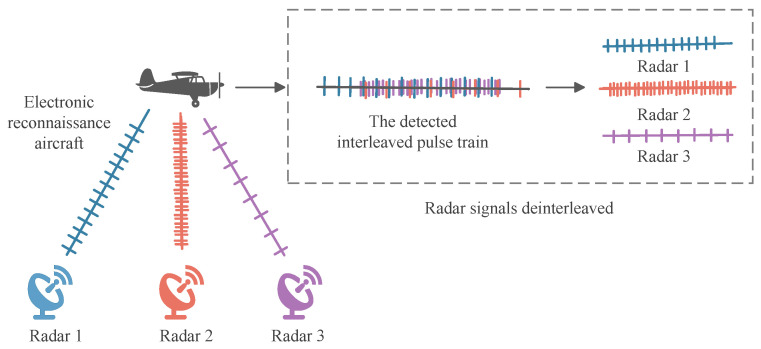
Radar Reconnaissance Aircraft Intercepting and Deinterleaving Signals Process.

The starting point of radar signal deinterleaving is the mixed pulse sequence intercepted by the electronic reconnaissance receiver. Figure 5 illustrates a reconnaissance aircraft performing its mission, receiving and then separating and classifying mixed signals from three radar emitters. Formally, this sequence can be modeled as a point process where each point represents a pulse event accompanied by a set of parameters describing its physical attributes.

Assume that within an observation time window *T*, a total of *N* pulses are intercepted. This mixed pulse sequence can be represented as

(1) $$P=\{p_{1},p_{2},\ldots ,p_{N}\}$$

where the pulses are ordered by their Time of Arrival (TOA). Each Pulse Description Word (PDW) $p_{i}$ is a multi-dimensional vector containing key measured parameters of that pulse, typically represented as:

(2) $$p_{i}=\left({\mathrm{TOA}}_{i},{\mathrm{DOA}}_{i},{\mathrm{RF}}_{i},{\mathrm{PW}}_{i},{\mathrm{PA}}_{i},\ldots \right)$$

**Figure 6:**
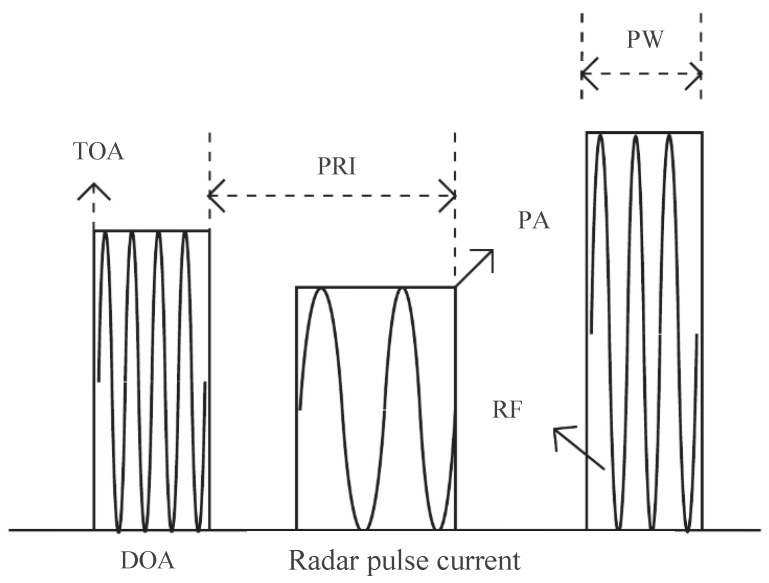
Physical Meanings of Parameters in a Pulse Description Word [36].

This parameter is summarized in Table 1 and presented in Figure 6. Here, ${\mathrm{TOA}}_{i}$ is the pulse arrival time, ${\mathrm{DOA}}_{i}$ is the direction of arrival, ${\mathrm{RF}}_{i}$ is the carrier frequency, ${\mathrm{PW}}_{i}$ is the pulse width, ${\mathrm{PA}}_{i}$ is the pulse amplitude, and so on.

Formally, let $S_{k}=\left\{p_{k,1},p_{k,2},\ldots ,p_{k,N_{k}}\right\}$ denote the pulse sequence emitted by the *k*-th radar, where $N_{k}$ is the number of pulses from emitter *k*. The intercepted pulse stream P corresponds to the set-theoretic union of all emitter sequences, temporally sorted by their Time of Arrival (TOA). This “interleaving” process can be mathematically expressed as:

(3) $$P=\overset{K}{\underset{k=1}{⋃}}S_{k}=\left\{p_{1},p_{2},\ldots ,p_{N}\right\}$$

subject to $\mathrm{TOA}\left(p_{n}\right)\leq \mathrm{TOA}\left(p_{n+1}\right)$ for all 1≤n<N, where $N=\sum_{k=1}^{K}N_{k}$ is the total number of intercepted pulses. The goal of deinterleaving is the inverse of this process, which is to partition the mixed set P into $K^{\prime }$ disjoint subsets $\left\{{\hat{S}}_{1},{\hat{S}}_{2},\ldots ,{\hat{S}}_{K^{\prime }}\right\}$, representing the estimated emitter sequences. Ideally, this partition satisfies:

(4) $$P=\overset{K^{\prime }}{\underset{j=1}{⋃}}{\hat{S}}_{j}\;\mathrm{and}\;{\hat{S}}_{i}\cap {\hat{S}}_{j}=\emptyset \;\mathrm{for}\;i\neq j$$

where each subset ${\hat{S}}_{j}$ is intended to approximate a true emitter sequence $S_{k}$.

The ideal deinterleaving result should ensure that the estimated number of emitters $K^{\prime }$ equals the true number *K*, and all pulses within each estimated subset ${\hat{S}}_{j}$ uniquely originate from a single true emitter sequence $S_{j}$. In practical scenarios, perfect partitioning is almost impossible due to signal loss, false pulses, and measurement errors. Furthermore, the number of emitters *K* is usually unknown and needs to be estimated by the algorithm, making deinterleaving a typical unsupervised learning problem, more specifically, a time series clustering or point process separation problem. The core challenge lies in the fact that the statistical characteristics of the source signals (individual radar pulse sequences), such as PRI patterns, are unknown and may change dynamically over time, while the mixing process (temporal overlap) introduces strong nonlinear interference. Table 2 lists the data in the simulation scenario. Each row is a PDW, that is, the vector of the pulse main parameter, and the last column is the corresponding pulse transmitter identifier to be found [37,38,39].

### 2.2. Analysis of Key Feature Parameters

#### 2.2.1. Inter-Pulse Feature Parameters

Inter-pulse feature parameters describe the macroscopic patterns of pulse sequences in dimensions such as time and space, serving as the core basis for traditional deinterleaving methods. Among these, Time of Arrival (TOA) is the most fundamental and important parameter. The TOA sequence itself contains all the temporal information of a radar emitter. By calculating the differences between TOAs, the Pulse Repetition Interval (PRI) is obtained, which is the time interval between the emission of consecutive pulses by a radar. For conventional radars, the PRI is usually a stable value or switches between a few values, forming its unique “temporal fingerprint”. Therefore, PRI extraction and analysis are critical steps in deinterleaving [40]. However, modern radars, in order to counter reconnaissance and interference, often employ complex PRI modulation schemes, such as PRI staggering, jittering, sliding, and grouped PRIs, posing significant challenges to methods based on stable PRI assumptions. Direction of Arrival (DOA) is another extremely important inter-pulse parameter, usually measured by an antenna array. Since different radar emitters are typically spatially separated, their DOA values should also exhibit distinct differences. Therefore, DOA is a very stable and reliable primary deinterleaving parameter that can serve as a “first line of defense” to initially partition signals from different spatial sectors, thereby greatly reducing the complexity of subsequent processing [41,42]. Even when multiple emitters exist in the same DOA direction, DOA can still serve as a strong constraint. The measurement accuracy and resolution of DOA directly affect deinterleaving performance, especially when dealing with multiple emitters with similar angles.

It is worth noting that recent advancements in data-driven and deep learning-based parameter estimation have significantly enhanced the quality of input features for deinterleaving systems. Novel approaches, such as joint estimation of DOA and frequency using uniform linear or rectangular arrays [43,44,45,46,47,48], and unfolding networks for decentralized estimation [49], provide robust spatial priors. These high-precision parameter estimates reduce feature ambiguity, thereby alleviating the burden on subsequent deinterleaving clustering algorithms.

#### 2.2.2. Intra-Pulse and Fingerprint Feature Parameters

Intra-pulse feature parameters describe the physical attributes within a single pulse and reflect the radar’s transmitter characteristics and operating modes. Radio Frequency (RF), or carrier frequency, is the operating frequency of the radar. For fixed-frequency radars, RF is a very stable deinterleaving basis. However, modern radars widely employ frequency agility techniques to enhance anti-jamming capabilities and detection performance, causing the RF to rapidly jump between pulses [32]. In such cases, the instantaneous RF value itself is no longer a stable clustering feature; instead, its hopping pattern (e.g., frequency hopping sequence) or frequency coverage range may become a new identification feature. Pulse Width (PW) and Pulse Amplitude (PA) also reflect the radar’s operating mode to some extent. For example, the PW in search mode and tracking mode might differ. PA is related to various factors such as radar transmit power, distance, and antenna pattern. Although it changes due to propagation path loss, statistically, the pulse amplitudes from the same emitter should follow similar distribution patterns [50].

With advancements in signal processing technology, an increasing number of intra-pulse modulation features are used for deinterleaving and identification, often referred to as “fingerprint” features because they can more finely characterize individual emitter differences. Examples include the modulation slope of Linear Frequency Modulated (LFM) signals, the coding sequence of phase-coded signals, and features of nonlinear frequency modulated signals [51,52,53]. Extracting these intra-pulse features requires more complex time-frequency or phase analysis of the pulse, incurring higher computational costs. However, they provide the possibility for fine-grained deinterleaving when conventional parameters (DOA, RF, PW) suffer from severe overlap, and are crucial for dealing with future complex signal environments.

### 2.3. Performance Evaluation Metrics

#### 2.3.1. Supervised Evaluation Metrics

During the research and simulation verification phases of algorithms, it is common to assume that the true emitter label (Ground Truth) for each pulse is known, allowing for quantitative evaluation of deinterleaving performance in a supervised manner. Deinterleaving Accuracy is the most intuitive metric, defined as the ratio of correctly assigned pulses to the total number of pulses. However, in cases of class imbalance (i.e., large differences in the number of pulses for different emitters), accuracy can be misleading. Therefore, more refined metrics such as Purity and Completeness are widely used. For a deinterleaved cluster, Purity measures what proportion of its members originate from the same true emitter, while Completeness measures what proportion of a true emitter’s members are correctly partitioned into the same cluster. Since these two metrics are often mutually constrained, their harmonic mean, the F1-Score, is typically used for comprehensive evaluation. In academia, the Rand Index (RI) and Adjusted Rand Index (ARI) are more favored evaluation standards. RI measures the similarity between the clustering result and the true labels by calculating the agreement of pairwise classifications. ARI is a corrected version of RI that introduces the expected RI value to eliminate the influence of random partitioning on the results, making the ARI range more meaningful (close to 1 indicates perfect match, close to 0 indicates random results). Consequently, it has become a de facto standard for evaluating clustering algorithm performance. Additionally, emitter count estimation accuracy is another important metric, measuring how closely the number of clusters detected by the algorithm approximates the true number of emitters.

#### 2.3.2. Unsupervised and Scenario-Based Evaluation Metrics

In practical application scenarios without true labels, unsupervised internal evaluation metrics are used to assess deinterleaving quality. These metrics are primarily based on the geometric properties of clusters, such as “cohesion” and “separation”. The Silhouette Coefficient is a commonly used metric that comprehensively considers the tightness of each sample to its own cluster and its separation from other clusters, with values ranging from [−1, 1]. Higher values indicate better clustering results [54]. The Davies–Bouldin Index (DBI) evaluates clustering effectiveness by calculating the ratio of intra-cluster scatter to inter-cluster distance for any two clusters; a lower DBI value indicates better performance. It should be noted that these internal metrics are based on certain assumptions (e.g., clusters are convex, uniformly dense), and may fail when dealing with complex shapes and multi-density clusters. Their evaluation results should only be used as a reference. Beyond clustering quality, scenario-based evaluation metrics are also crucial. Computational complexity and time efficiency are key metrics for evaluating whether an algorithm can meet real-time requirements. For instance, for ESM systems requiring online processing, deinterleaving algorithms must complete processing within a short time after pulse arrival. Robustness is another important aspect, including adaptability to pulse loss rate, false alarm rate, parameter measurement errors, and varying signal-to-noise ratios. An excellent algorithm should maintain relatively stable performance under various adverse conditions.

## 3. Traditional Radar Signal Deinterleaving Methods

Traditional deinterleaving methods form the cornerstone of intelligent radar signal processing. Their design philosophies and limitations provide important insights for the evolution of subsequent methods. This chapter categorizes traditional methods into three main groups: those based on PRI temporal features, those based on multi-dimensional parameter space clustering, and hybrid and heuristic approaches, with in-depth analysis of specific techniques within each category.

### 3.1. PRI Temporal Feature-Based Methods

As one of the most important and stable features of radar signals, PRI has been the core basis for signal deinterleaving for a considerable period. The fundamental idea behind these methods is to extract the implicit PRI pattern by analyzing the Time of Arrival (TOA) sequence, thereby classifying pulses belonging to the same PRI pattern into one group.

#### 3.1.1. PRI Search Based on Difference Histograms

Histogram-based methods are among the earliest and most classic PRI search techniques. Their basic principle is to statistically analyze the distribution of TOA differences between pulses; the peak position in the histogram indicates a potential true PRI value. Typical representatives include the Cumulative Difference of Inter-Pulse Intervals (CDIF) histogram and the Sequential Difference of Inter-Pulse Intervals (SDIF) histogram.

The core idea of the CDIF algorithm is “brute-force” search. It calculates the TOA differences which can be expressed discretely as:

(5) $$\Delta t={\mathrm{TOA}}_{j}-{\mathrm{TOA}}_{i}$$

for all possible pulse pairs ($p_{i},p_{j}$) in the received pulse sequence and then quantizes these differences into “bins” to form a histogram [8]. If a stable radar signal with PRI $T_{0}$ exists in the sequence, then the TOA difference between its pulses will inevitably be an integer multiple of $T_{0}$, i.e.,

(6) $$\Delta t=k\cdot T_{0}$$

Consequently, in the histogram, positions $T_{0},2T_{0},3T_{0},\ldots$ will form distinct peaks due to the accumulation of differences. By detecting these harmonic peaks, potential PRI values can be estimated. The advantages of CDIF lie in its simple and intuitive principle, and its utilization of all pulse pair information provides good robustness against a small number of pulse losses. However, its disadvantages are equally significant. Firstly, its computational complexity is $O(N^{2})$, making it computationally infeasible when the pulse density *N* is large. Secondly, and more critically, CDIF generates a large number of “false peaks”. These false peaks arise from the interactions between pulses from different emitters and severely interfere with the detection of true PRI peaks. Moreover, the presence of harmonics complicates the determination of the fundamental PRI. To mitigate these issues, various improvement strategies have been proposed. For instance, early attempts involved dynamic thresholding techniques to suppress false peaks based on statistical priors. However, even these optimized histogram-based approaches fundamentally fail when encountering modern complex PRI modulations, such as Dwell-and-Switch patterns, due to their reliance on fixed statistical assumptions.

**Figure 7:**
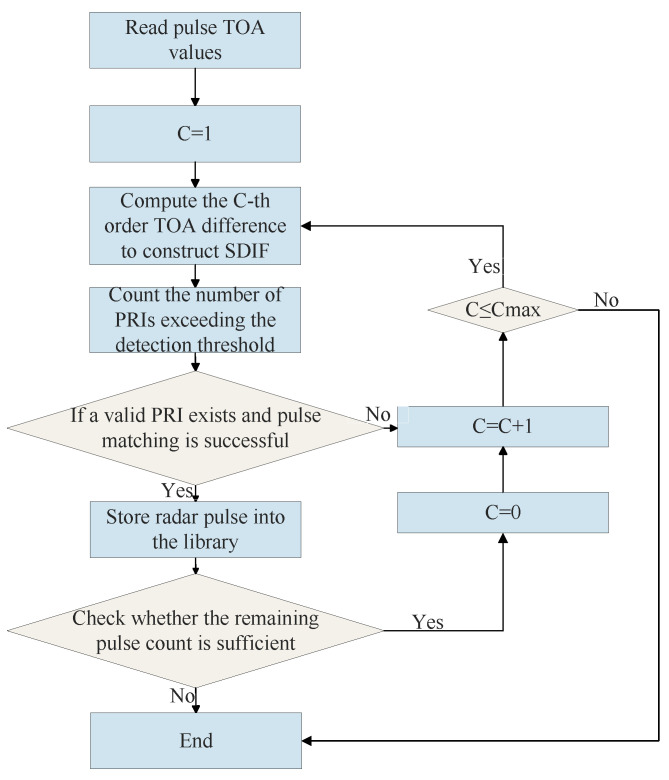
Operational Flowchart of the Sequential Difference Histogram (SDIF) Algorithm.

The SDIF algorithm improves upon CDIF in terms of computational efficiency. The detailed operational flow of SDIF method is illustrated in Figure 7 below. Instead of calculating differences for all pulse pairs, it only computes differences between adjacent or near-adjacent pulses [9]. For example, first-order SDIF only computes differences between ($p_{i+1},p_{i}$), while second-order SDIF computes differences between ($p_{i+2},p_{i}$). This reduces its computational complexity to linear O(N·M) (where M is the order of the difference), significantly improving processing speed. Concurrently, by reducing the range of pulse pairs considered, the false peak problem is also mitigated to some extent. However, SDIF becomes extremely sensitive to pulse loss. Once a pulse is lost in the sequence, all subsequent difference calculations based on that lost pulse will be erroneous, rendering PRI extraction impossible.

Efforts have been made to improve SDIF algorithms by integrating fault-tolerant mechanisms [9]. Specifically, this approach applies the principle of analyzing the SDIF failure mechanism under RF stealth conditions to design more resilient statistical processing steps. In summary, both CDIF and SDIF are based on the strong assumption of stable or simply varying PRIs. For the complex PRI modulations (e.g., agility, jitter, sliding) commonly employed by modern radars, these histogram-based statistical methods fail completely due to their inability to form clear peaks [40,55].

#### 3.1.2. PRI Extraction Based on Transform Domain Methods

To better extract periodic features from mixed TOA sequences, researchers have borrowed ideas from signal processing and transformed the PRI search problem into analysis in the transform domain. The core idea behind this approach is to explicitly manifest the implicit periodic patterns in the time domain as energy peaks in the transform domain through a mathematical transformation, thereby enhancing detection robustness and accuracy. PRI Transform and Hough Transform are two representative techniques.

PRI Transform is a technique specifically designed for periodic detection of pulse sequences [56]. The basic idea is similar to Fourier Transform in signal processing: a discrete TOA sequence $t_{1},t_{2},\ldots ,t_{N}$ is treated as a series of impulse functions on the time axis. It is then mapped to the PRI domain by performing an operation with a kernel function containing PRI harmonic information. In the PRI domain, the horizontal axis represents candidate PRI values, and the vertical axis represents the “energy” or “likelihood” of that PRI value. If the original sequence contains a PRI value, it will manifest as a sharp peak in the PRI spectrum. Compared to CDIF, PRI Transform effectively suppresses the generation of false peaks through comprehensive utilization of harmonics and exhibits stronger resolution for harmonics [40]. However, the performance of PRI Transform is affected by the selection of transform parameters (e.g., PRI search range, resolution), and its physical meaning is less intuitive than the histogram method. Crucially, it is fundamentally designed for detecting fixed periods. For non-stationary PRI sequences (e.g., sliding PRIs), the transformation results in spectral broadening and energy leakage, which complicates peak detection.

The Hough Transform, originally a classic algorithm in image processing for detecting shapes like lines and circles, was later applied to radar signal deinterleaving. Its core idea is a “voting” mechanism. For a pulse sequence with PRI $T_{0}$ and initial phase (or reference time) $t_{ref}$, the TOA of the *n*-th pulse can be expressed as:

(7) $$t_{n}=n\cdot T_{0}+t_{ref}$$

This is a parametric equation with respect to ($T_{0},t_{ref}$). The Hough Transform maps each pulse’s TOA value from the time domain space to the ($T_{0},t_{ref}$) parameter space. Each pulse point in the time domain corresponds to a line or curve in the parameter space. If multiple pulses lie on the same line (i.e., belong to the same PRI sequence), the curves corresponding to these pulses in the parameter space will intersect at a point. By setting accumulators in the parameter space and counting the intersections of curves, peaks are formed. The location of a peak corresponds to the estimated PRI and phase. The greatest advantage of the Hough Transform is its strong robustness against pulse loss and noise (false pulses) because it utilizes the global linear relationship of pulses; the absence or addition of individual points does not affect the overall trend. However, its computational cost and memory requirements are enormous, especially when the PRI range is wide and high resolution is required, causing the parameter space to expand dramatically in dimension and size. Furthermore, the standard Hough Transform is primarily applicable to fixed PRIs or simple linearly varying PRIs. For complex nonlinear PRI modulations, the mapping relationship becomes complicated, making direct application difficult [57].

#### 3.1.3. Methods Based on Temporal Correlation and Sequence Search

In addition to statistical and transform-based methods, another category of methods extracts PRI sequences by directly performing correlation and search in the time domain. These methods are generally more heuristic, attempting to mimic the human thought process of analyzing pulse sequences. Sequence search is a typical representative.

The basic idea is as follows: starting with a pulse, search for subsequent pulses that satisfy a specific PRI tolerance and incrementally extend this pulse chain. For example, starting with pulse $p_{i}$, guess a PRI value (possibly from a rough SDIF histogram) and then search for the existence of a pulse $p_{j}$ around ${\mathrm{TOA}}_{i}+\mathrm{PRI}$. If found, take $p_{j}$ as the new starting point and continue searching around ${\mathrm{TOA}}_{j}+\mathrm{PRI}$. This process repeats. When the pulse chain extends to a certain length, a potential emitter sequence is considered to be found. The advantage of this method is its intuitiveness, and it can directly handle some simple PRI modulations, such as staggered PRIs (by simultaneously searching for multiple PRI values). However, its computational complexity is very high, approaching exhaustive search, especially in scenarios with high pulse density and a wide range of candidate PRIs. Furthermore, it is highly sensitive to the starting point and the initial PRI guess, easily falling into local optima or generating numerous erroneous pulse chains. To improve efficiency and robustness, researchers have proposed correlation analysis methods [58,59]. For instance, for each pulse, a “neighboring pulse feature vector” can be constructed, containing relative TOA information of other pulses within a certain time window before and after it. Then, by calculating the correlation between the feature vectors of different pulses, it can be determined whether they belong to the same PRI pattern. If the neighboring pulse distribution patterns of two pulses are highly similar, they likely originate from the same emitter. Wang [58] and Zhou [59] et al. respectively studied deinterleaving algorithms based on correlation analysis and correlation distance differencing.

The advantage of these methods is their ability to utilize local temporal structural information of pulses, offering some tolerance to PRI jitter. However, their performance is highly dependent on the design of the feature vectors and the choice of correlation metric function, and discrimination of correlation becomes ambiguous and difficult when dealing with multiple complex PRI modulations interleaved.

#### 3.1.4. Summary of PRI Methods

While the aforementioned methods form the foundation of deinterleaving, their performance boundaries differ significantly, as summarized in Table 3.

- CDIF and SDIF:These histogram-based methods are computationally efficient (O(N) to $O(N^{2})$) but fundamentally rely on the periodicity assumption. They typically fail when the Pulse Repetition Interval (PRI) jitter exceeds 10–15% of the mean value, as the histogram peaks become smeared and indistinguishable from noise. Furthermore, in high-density environments (e.g., >$10^{5}$ pulses/sec), the accumulation of harmonic “ghost peaks” often masks the true emitter PRI.
- Hough Transform: This approach offers superior robustness against missing pulses, maintaining effectiveness even with pulse loss rates exceeding 30–40% due to its global voting mechanism. However, its primary bottleneck is the computational explosion when handling agile PRIs. The parameter space must be discretized finely; thus, for complex modulation types, the search space becomes prohibitively large for real-time applications.
- Sequential Search: Unlike global statistical methods, this approach relies on local pulse matching. Its critical failure boundary lies in error propagation and computational complexity under high density. Specifically, if the initial pulse pair selection is incorrect (due to noise or overlapping pulses from other emitters), the error propagates through the entire chain, leading to false tracks. Additionally, in environments with high pulse density (e.g., >$10^{5}$ pulses/sec), the combinatorial burden of checking candidate pulses against potential PRI criteria renders it too slow for real-time processing, although it tolerates moderate PRI jitter (approximately 5–10%) via tolerance windows.

### 3.2. Multi-Dimensional Parameter Space Clustering-Based Methods

When PRI features become unstable and unreliable due to complex modulations, jointly utilizing multiple inter-pulse macroscopic features (e.g., DOA, RF, PW) from PDWs for deinterleaving becomes a necessary choice. Clustering-based methods treat each pulse as a point in a multi-dimensional feature space, transforming the deinterleaving problem into a classic unsupervised clustering problem.

It is noteworthy that although methods in this stage incorporate more parameter dimensions, their essence remains based on macroscopic similarity between pulses, with limited capability to mine and utilize intra-pulse fingerprint features. Nevertheless, advancements in multidimensional signal processing continue to refine this approach. For instance, tensor-based data-driven methods for multidimensional harmonic retrieval [60,61] have demonstrated efficacy in complex channel sounding scenarios. These techniques offer a rigorous theoretical framework for modeling the multidimensional structure of interleaved signals, providing higher-quality feature inputs for the clustering algorithms discussed below.

#### 3.2.1. Partition-Based Clustering Paradigm

Partition-based clustering methods attempt to directly partition the dataset into *K* non-overlapping clusters, where *K* is a pre-specified hyperparameter. The K-Means algorithm is the most well-known and widely used method in this category [62].

Its algorithmic flow is very intuitive: first, randomly select *K* data points as initial cluster centers (centroids); then, enter an iterative process consisting of two steps: (a) assignment step, where each data point is assigned to the centroid closest to it; (b) update step, where the centroid of each cluster is recalculated, typically as the mean of all data points within that cluster. This iterative process continues until the cluster assignments no longer change or the centroid positions stabilize. The advantages of K-Means lie in its simple principle, ease of implementation, and fast convergence, making it efficient for processing large-scale datasets. However, its inherent drawbacks are significantly amplified in the specific application of radar signal deinterleaving. First and foremost, the core limitation is the need to pre-specify the number of clusters *K*. In non-cooperative electronic reconnaissance scenarios, the number of emitters is unknown, making the choice of *K* a tricky issue. Second, K-Means is extremely sensitive to the initial centroid selection; different initial points can lead to entirely different clustering results, and the algorithm easily falls into local optima. Third, K-Means is based on Euclidean distance for partitioning, implicitly assuming that clusters are convex, spherical, and of similar size. Its clustering performance will be poor for radar signal pulses whose parameter distributions exhibit non-convex shapes or vary in size. Finally, since centroids are calculated via the mean, K-Means is very sensitive to noise and outliers.

Numerous improved algorithms have been proposed to overcome these shortcomings. For example, K-Medoids (or PAM) algorithm uses actual data points (medoids) within clusters as centroids instead of means, greatly enhancing robustness to noise and outliers. Furthermore, more research has focused on combining K-Means with other techniques to form hybrid algorithms. For instance, combining it with the Canopy algorithm [63] for a fast, rough pre-clustering to estimate *K* and initial centers; or utilizing intelligent optimization algorithms (e.g., Ant Colony Optimization) to search for optimal cluster centers. Tang et al. also proposed an improved K-Means pre-selection method [11]. Although these improvements have alleviated some of K-Means’ problems to a certain extent, they have not fundamentally addressed the core limitations of needing to pre-set *K* and the strong assumption about cluster shapes.

#### 3.2.2. Density-Based Clustering Paradigm

Unlike partition-based methods that forcefully assign all points to a cluster, density-based clustering methods start from the distribution density of data points. Their core idea is that a cluster is composed of points in high-density regions, separated by low-density regions.

**Figure 8:**
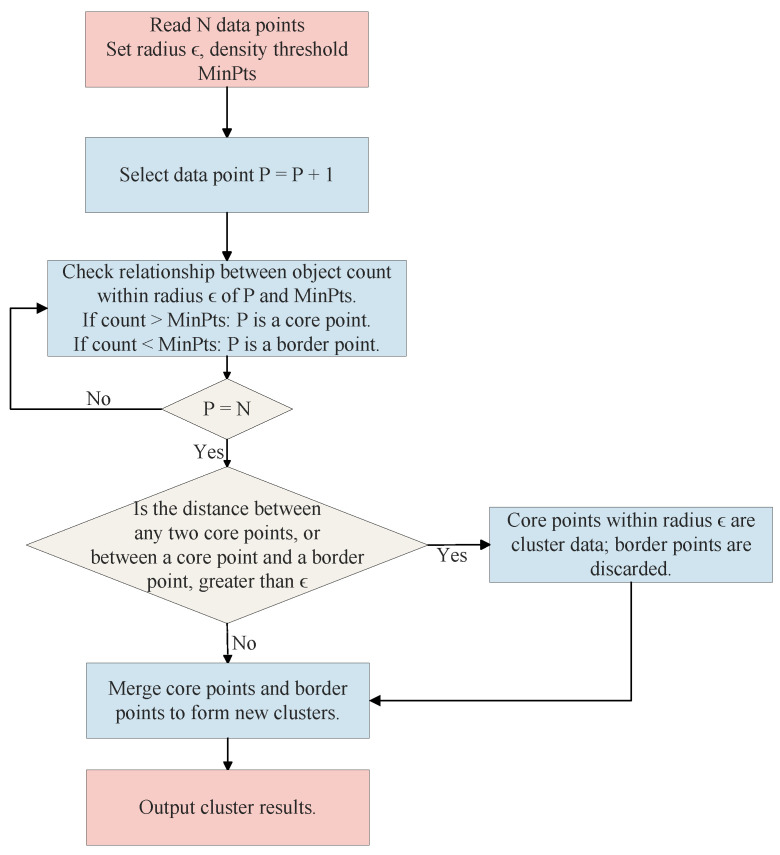
Flowchart of the DBSCAN clustering algorithm.

DBSCAN (Density-Based Spatial Clustering of Applications with Noise) is a milestone algorithm in this field [10,54,64]. The flowchart of the DBSCAN algorithm is shown in Figure 8. The algorithmic logic of DBSCAN revolves around two core parameters: neighborhood radius (Eps) and minimum number of points in the neighborhood (MinPts). Based on these two parameters, it classifies points in the dataset into three types: core points, border points, and noise points. The algorithm starts from an arbitrary core point and expands outward through “density-reachability,” partitioning all density-connected points into a cluster. A key advantage of DBSCAN is its ability to automatically determine the number of clusters based on data distribution, without requiring it as a preset parameter. This characteristic makes it exceptionally well-suited for deinterleaving scenarios where the number of emitters is unknown a priori. Furthermore, due to its expansion based on connectivity, DBSCAN can discover clusters of arbitrary shapes (non-convex) and identify low-density region points as noise, thus effectively handling anomalous pulses [10,64]. However, the successful application of standard DBSCAN heavily relies on the appropriate setting of the Eps and MinPts parameters.

Numerous research efforts have been dedicated to adaptive parameter selection [54,65,66]. For example, Su et al. proposed an adaptive DBSCAN algorithm based on a novel Cluster Validity Index (CVI) [54]. Another major limitation of DBSCAN is its difficulty in handling datasets with uneven densities. Because it uses a globally uniform density threshold to measure all regions, a single parameter setting cannot adapt to multiple clusters with significantly different densities. To address this, more advanced density clustering algorithms such as OPTICS and HDBSCAN have been proposed. Wei et al. directly targeted the radar signal scenario and proposed an improved adaptive multi-density DBSCAN method to better adapt to complex electromagnetic environments [65].

#### 3.2.3. Graph Theory and Hierarchical Clustering Paradigms

In addition to mainstream partition-based and density-based clustering, other clustering techniques have also been applied to radar signal deinterleaving, modeling data structures from different perspectives.

Hierarchical Clustering is an important category among these methods [67,68]. Unlike K-Means, which completes partitioning in one step, hierarchical clustering builds a nested hierarchy of clusters (often represented as a dendrogram) for clustering. It can be either agglomerative (bottom-up) or divisive (top-down). The advantage of hierarchical clustering is that it does not require pre-setting the number of clusters; users can “cut” the dendrogram at different levels to obtain different numbers of clusters. However, its computational complexity is usually high (at least $O(N^{2})$), and once a merge or split operation is completed, it cannot be undone, potentially leading to suboptimal solutions.

Spectral Clustering is a powerful graph theory-based clustering method [69]. It views data points as vertices of a graph, with the similarity between points as edge weights. The clustering problem is then cleverly transformed into a graph partitioning problem, aiming to find a cutting strategy that minimizes the weights of edges between different subgraphs while maximizing the weights of edges within subgraphs. This is typically achieved through eigenvalue decomposition of the graph’s Laplacian matrix. The main advantage of spectral clustering is its ability to easily identify non-convex clusters and its lack of strong assumptions about data distribution. Li et al. proposed a deinterleaving method based on SOM anchor extraction and graph fusion using spectral clustering [69]. Its main challenges lie in the construction of the similarity matrix and the computational cost of eigenvalue decomposition. A summary of these clustering-based methods is provided in Table 4.

### 3.3. Hybrid and Heuristic Methods

To overcome the limitations of single methods, many research efforts focus on fusing different traditional methods or introducing physical models and heuristic rules to form hybrid deinterleaving strategies.

#### 3.3.1. Combination of PRI Preprocessing and Clustering

This is a very common and effective hybrid strategy. The core idea is to first “purify” or “tag” the pulse sequence using PRI analysis techniques, and then feed the processed results or extracted PRI features into a clustering algorithm for fine-grained partitioning. For example, one could first run SDIF or PRI Transform to identify some high-confidence PRI values. Then, these PRI values are used as prior knowledge to guide the clustering process. One approach is to add whether a pulse conforms to a certain PRI pattern as a new feature dimension to the feature vector fed into the clustering algorithm. Another more direct approach is to first separate the pulse sequences with stable PRIs (the “easy” parts) using the extracted PRI values, and then perform clustering only on the remaining, more complex pulses. This “divide and conquer” strategy can effectively reduce the complexity and ambiguity of subsequent clustering tasks. Zhang Yixiao et al. studied a deinterleaving method combining PRI Transform and data field with clustering, first obtaining PRI information using PRI Transform, then clustering based on data field theory, achieving better results than single methods. The advantage of this hybrid method is its comprehensive utilization of both temporal and multi-dimensional parameter information of pulses, achieving complementary strengths. However, its performance is highly dependent on the accuracy of the PRI preprocessing step. If the PRI extraction stage makes errors and produces incorrect PRI estimates, these errors will be carried into the subsequent clustering process, potentially leading to worse results than not using PRI preprocessing.

#### 3.3.2. Heuristic Methods Based on Data Field and Physical Models

The Data Field theory borrows ideas from physics field theory [70,71]. It treats each data point (pulse) as a field source that generates a scalar field in its surrounding space, with field strength inversely proportional to distance. The superposition of fields generated by the entire dataset forms a data field. In this data field, regions with high data point density form “potential” peaks, while sparse regions form “potential” troughs. Therefore, cluster centers can be considered as points of maximum potential in the data field’s potential function. By finding these peak points, cluster centers and quantities can be determined. The advantage of the data field method is that it does not require pre-specifying the number of clusters and has no strict requirements on cluster shapes. Guo Qiang et al. combined the data field with cloud models for deinterleaving multi-mode radar signals [71]. Beyond data fields, other heuristic methods attempt to incorporate more physical or domain knowledge. For instance, when processing antenna scanning signals, a mathematical model describing the variation of pulse amplitude over time (e.g., a Gaussian function model) can be established, and subsequent fitting of this model can be used to separate pulses belonging to the same scanning period. The advantage of these methods is their good physical interpretability and their effectiveness in specific scenarios. However, their disadvantage is poor generality, being highly dependent on the accuracy of the prior model. When the actual signals do not conform to the pre-set model, their performance will degrade sharply.

#### 3.3.3. Methods Based on Decision Trees and Rule Inference

In the early stages of artificial intelligence development, methods based on expert systems and rule inference were also used for radar signal deinterleaving. The core of these methods is to build a rule base containing numerous “IF-THEN” rules summarized by radar experts. For example, “IF DOA is within a small range AND RF is within a small range AND PRI is stable THEN these pulses belong to the same emitter”. The deinterleaving process is a logical inference process where the system matches received pulse parameters with rules in the rule base and makes decisions based on the matching results. Decision trees are a common tool for implementing this rule inference. By training on a large amount of sample data, a decision tree for classification can be automatically learned. Each internal node of the tree represents a judgment on pulse parameters (e.g., “RF > 10 GHz”), and each leaf node represents a deinterleaving result (belonging to a certain emitter). The advantage of these methods is their intuitive model and ease of understanding and explanation; their decision process is transparent. However, their disadvantages are also very apparent. Firstly, rule formulation relies heavily on expert knowledge, which is time-consuming and difficult to cover all complex situations. Secondly, rules based on hard thresholds are very sensitive to parameter measurement errors and jitter. Lastly, when facing high-density, highly overlapped complex electromagnetic environments, simple rules are prone to conflict and failure, leading to poor deinterleaving performance. Therefore, with the rise of machine learning, especially statistical learning and deep learning, these symbolic logic inference-based methods have gradually been marginalized in modern signal deinterleaving research.

## 4. Deep Learning-Based Radar Signal Deinterleaving

The efficacy of the deep learning models discussed in this section is intrinsically linked to the quality and quantity of training data. While a comprehensive review of modern open-source radar datasets (such as EBDSC) is reserved for Section 5 to maintain the chronological narrative, this section focuses on the architectural innovations and learning strategies. Readers interested in the specific benchmarks enabling these methods are encouraged to refer to Section 5 following this discussion.

Traditional methods, when faced with modern complex electromagnetic environments, have limited adaptability and generalization capabilities due to their reliance on manually designed rules or fixed mathematical models. Deep Learning (DL) methods differ fundamentally from traditional methods in that they abandon the reliance on manually designed features and explicit rules. Through an end-to-end learning paradigm, they can automatically and hierarchically learn and fuse inter-pulse temporal patterns and intra-pulse fingerprint features from data. This powerful feature representation capability allows them to demonstrate significant advantages in addressing modern complex electromagnetic environments.

This chapter systematically elaborates on the specific applications, core ideas, and evolutionary advancements of mainstream deep learning models in the deinterleaving task, organized by model categories.

While this section focuses on deinterleaving, the paradigm shift towards data-driven methodologies is evident across the broader spectrum of radar signal processing. For instance, deep learning has demonstrated remarkable success in distributed unmanned system direction finding [72], high-resolution automotive radar imaging [73], and unbiased estimation in phased array regimes [74]. These achievements provide a solid theoretical and practical foundation for migrating similar data-driven strategies to the pulse deinterleaving domain.

### 4.1. Sequence Modeling Based on Recurrent Neural Networks (RNNs)

#### 4.1.1. Basic RNN Model and Its Application in Sequence Labeling

Recurrent Neural Networks (RNNs) and their variants are neural network architectures designed for processing sequential data, with the core idea of utilizing internal recurrent connections to capture temporal dependencies within sequences. Since radar pulse sequences inherently possess temporal characteristics, modeling the deinterleaving problem as a sequence labeling task and using RNNs for processing is a very intuitive and effective approach. Standard RNNs suffer from severe gradient vanishing or exploding problems when processing long sequences, making it difficult for them to learn long-term dependency patterns. To address this issue, Long Short-Term Memory (LSTM) and Gated Recurrent Unit (GRU) [75,76] were proposed and have become mainstream choices for sequence processing tasks. LSTMs, by introducing three sophisticated “gate” structures—input gate, forget gate, and output gate—along with an independent “cell state,” effectively control the flow and storage of information, thereby mitigating gradient problems and enabling the learning of longer-term temporal dependencies [77,78,79]. In radar signal deinterleaving, the PDW sequence (typically a TOA sequence or a multi-dimensional parameter sequence) can be input to the LSTM network pulse by pulse. The network outputs a probability distribution at each time step, indicating the probability of the current pulse belonging to each predefined emitter. Luo et al. validated the feasibility of using LSTM for signal deinterleaving and individual identification, achieving a deinterleaving accuracy of 87% in pairwise mixtures of five distinct emitters with pulse loss rates ranging from 0% to 30% [77]. The essence of this method is to treat deinterleaving as a multi-class classification problem, classifying pulses at each time step. However, this simple point-wise classification ignores the structural relationships between pulses, especially for patterns like PRI that require multiple preceding pulses to determine.

#### 4.1.2. Bidirectional RNNs and Context Information Fusion

To further enhance the model’s contextual understanding capabilities, Bidirectional RNNs (Bi-RNNs), particularly Bidirectional LSTMs (Bi-LSTMs), are widely adopted [80]. Standard unidirectional RNNs, when processing input at time step t, only have hidden states containing information up to time t, which is insufficient for many tasks. For example, when understanding the meaning of a sentence, the meaning of a word depends not only on the preceding words but also on the subsequent words. Similarly, in radar signal deinterleaving, the determination of a pulse’s affiliation should also rely on its preceding and succeeding pulse context. Bi-LSTMs perfectly address this problem. They consist of a forward LSTM that processes the sequence in chronological order and a backward LSTM that processes the sequence in reverse chronological order. At each time step t, the forward LSTM outputs a hidden state $h_{\mathrm{forward},t}$ based on past information, and the backward LSTM outputs a hidden state $h_{\mathrm{backward},t}$ based on future information. These two hidden states are concatenated $\left[h_{\mathrm{forward},t},h_{\mathrm{backward},t}\right]$ as the final representation for that time step. This allows the model to simultaneously consider complete preceding (“past”) and succeeding (“future”) context information when making a decision about the current pulse. This is crucial for accurately determining a pulse’s affiliation, as PRI relationships are bidirectional; a pulse’s PRI depends not only on the pulse preceding it but also on the pulse succeeding it. For instance, to confirm if a pulse belongs to a sequence with PRI T, one must check not only if there is a pulse at time T before it but also if there is a pulse at time T after it. The incorporation of Bi-LSTM enables the model to better capture these bidirectional dependencies, thereby demonstrating enhanced robustness when processing complex patterns such as PRI staggering and jitter. Furthermore, an accuracy of 72.74% was achieved under conditions of a 30% pulse missing rate and a 30% spurious pulse ratio [78].

#### 4.1.3. RNN Models Enhanced by Attention Mechanisms

Although Bi-LSTMs can fuse complete contextual information, they process all contextual information uniformly. However, in practice, the importance of different context pulses varies when judging the affiliation of the current pulse. The introduction of the Attention Mechanism is precisely to solve this problem, empowering the model with the ability to “focus” on key information [78,81]. By adding an attention layer on top of Bi-LSTM, the model can perform a weighted aggregation of Bi-LSTM outputs from all time steps before outputting the final pulse label. This means that when judging the affiliation of pulse A, the model can not only see the context of the entire sequence but also automatically learn which pulses in the sequence (e.g., pulses B and C that form a stable PRI relationship with pulse A) are most important for its decision, assigning them higher weights while assigning lower weights to irrelevant interfering pulses. This weight is dynamically calculated and depends on the relationship between the currently focused pulse and other pulses in the context. The IA-Bi-LSTM deinterleaving model, proposed by Zhang Yuxiang et al., leverages this mechanism to enhance the capture of complex PRI patterns [78]. IA-Bi-LSTM demonstrated superior performance over traditional LSTM and Bi-LSTM methods under various interference, missing, and jitter conditions. Tested on datasets involving 2 and 3 radars, the novel method exhibited significant robustness and high accuracy in both single interference environments and extreme composite conditions. Its advantages were particularly evident in scenarios with severe pulse missing rates, spurious pulse ratios, and pulse jitter rates. Under a 30% missing rate and a 30% spurious pulse ratio, the accuracy reached 74.84%. The introduction of the attention mechanism also improves the interpretability of RNN models. By visualizing the distribution of attention weights, we can intuitively see which information the model relies on most when making decisions. This not only enhances model performance but also provides some understanding of the model’s internal workings. Therefore, RNNs and their variants are well-suited for modeling the inter-pulse temporal dependencies of radar signals, especially for capturing core sequential patterns like PRI.

### 4.2. Global Relationship Modeling Based on Transformers

#### 4.2.1. Transformer Architecture and Self-Attention Mechanism

**Figure 9:**
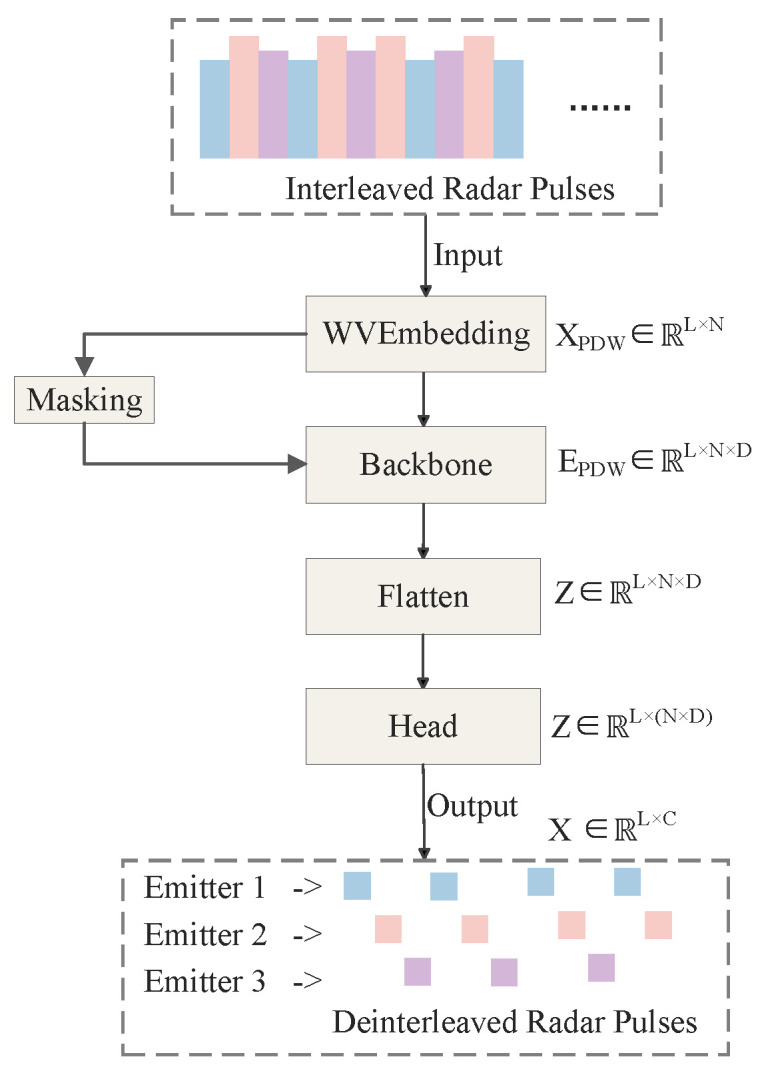
The overall architecture diagram of the RSS model, with the main structure being stacked ModernTCN blocks [82].

To overcome the parallelism limitations of RNNs and the challenges of capturing long-range dependencies, the Transformer model emerged and achieved revolutionary success in natural language processing [81]. Figure 9 shows the complete structure of the Transformer, which consists of two parts: an encoder and a decoder. The encoder’s role is to map input data to a feature space, while the decoder utilizes these features to reconstruct the original sequence. The encoder and decoder contain multi-head attention mechanisms, feed-forward neural networks, and residual connections. The multi-head attention mechanism is responsible for extracting parallel features from input data, while the positional encoding mechanism records positional information, ensuring feature relevance. The core innovation of the Transformer model is its complete abandonment of RNN’s recurrent structure, relying solely on the Self-Attention mechanism to model global dependencies within sequences. The core idea of the self-attention mechanism is that the updated representation of one element in a sequence is a weighted sum of the representations of all elements in the sequence, and this weight is dynamically calculated based on the “relevance” or “compatibility” between that element and all other elements. Specifically, for each pulse in the sequence, the model generates three vectors: Query (Q), Key (K), and Value (V). Then, each pulse’s Q vector is used to calculate the dot product similarity with all pulses’ K vectors. After scaling and softmax normalization, the attention weights of this pulse relative to all other pulses are obtained. Finally, the V vectors of all pulses are weighted and summed according to these weights to obtain the new representation of that pulse. This process is computed simultaneously and in parallel for all pulses in the sequence, resulting in significantly higher computational efficiency than RNNs. To enhance the model’s expressive power, Transformer also introduces Multi-Head Attention, which means running multiple self-attention modules in parallel and concatenating their results. This allows the model to simultaneously attend to information from different positions in different representation subspaces.

#### 4.2.2. Pulse Sequence Deinterleaving Based on the Encoder

In radar signal deinterleaving, the global relationship modeling capability of the Transformer makes it highly suitable for discovering complex, non-contiguous PRI patterns (e.g., grouped PRI, jittered PRI) [83,84]. A pulse can directly interact with any pulse in the sequence that may have a PRI relationship with it through the self-attention mechanism, regardless of their distance. Standard Transformer deinterleaving models typically employ its encoder part. The encoder consists of multiple identical stacked layers, each containing a multi-head self-attention layer and a feed-forward neural network layer, supplemented by residual connections and layer normalization. The input pulse sequence (each pulse being a feature vector) is first passed through a positional encoding layer to inject positional information (as the self-attention mechanism itself does not contain sequential information), and then fed into the encoder stack. After processing by multiple encoder layers, the model generates a representation vector for each input pulse that is deeply fused with global contextual information. Finally, a classification layer (e.g., Softmax) is attached on top of these representation vectors to predict the emitter label for each pulse. Zhou et al. [83] proposed a Transformer network based on a multi-self-attention coupling mechanism, specifically designed for radar signal deinterleaving. Experimental results demonstrated that for the deinterleaving of four radars, an accuracy exceeding 89% was achieved even under an 80% pulse loss rate, outperforming traditional LSTM models. Liu et al. also explored the application of Transformers in deinterleaving tasks and achieved positive results [84].

#### 4.2.3. Non-Recurrent Convolutional Architectures: TCN and Its Variants

Besides Transformers, Temporal Convolutional Networks (TCNs) are another noteworthy non-recurrent sequence processing architecture that competes with RNNs. The core of TCN lies in Causal Convolutions, a special type of 1D convolution that ensures that when predicting the output at time step t, only information up to time t is used, and “future” information is not accessed, thus preserving temporal causality. To capture long-term dependencies, standard convolutions require a very large number of layers, making them difficult to train. TCN cleverly solves this problem through Dilated Convolutions. Dilated convolutions allow the convolutional kernel to sample the input at specified intervals (dilation rates), thereby exponentially expanding the receptive field of each layer without increasing the number of parameters. By stacking multiple layers of dilated convolutional layers with exponentially increasing dilation rates [85,86], TCN can achieve a very large receptive field with a relatively shallow network structure, enabling it to capture long-range dependencies. Furthermore, TCN extensively uses Residual Connections, which are crucial for building deep networks and preventing gradient vanishing. Compared to RNNs, TCN computations are fully parallelized because they are based on convolutional operations. Compared to Transformers, TCNs typically have lower computational costs and memory footprints because they process local dependencies (although the receptive field can be large), rather than global ones. Wang et al. proposed a deep learning deinterleaving method based on a temporal convolutional attention network and subsequently published more in-depth research, demonstrating the effectiveness of TCN in processing intercepted radar pulse streams. TCN offers an efficient alternative for sequential deinterleaving that combines parallel computation capability with powerful temporal modeling power.

To further explore the potential of TCN in complex temporal pattern recognition, researchers have begun optimizing its input representation and output mechanisms to better align with the intrinsic challenges of radar signal deinterleaving. In recent research, Hu et al. [82] proposed a TCN deinterleaving method enhanced by Wavelet Embeddings (WVEmbs) and a dynamic masking mechanism. The overall architecture diagram is shown as follows. In a complex test scenario with 12 types of interleaved radars from the second “Sharp Eyes” Electromagnetic Big Data Challenge dataset, this method achieved deinterleaving accuracies (Accuracy) exceeding 88.68%, significantly outperforming baseline models including standard TCN and Transformer. Its core innovations lie in two aspects: First, addressing the characteristic of radar PRI patterns often having multi-scale and multi-resolution properties (e.g., macroscopic trends in sliding PRI and microscopic variations in jittered PRI), they abandoned traditional fixed positional encodings and adopted wavelet embeddings to encode the input pulse sequence. The multi-scale analysis capability of wavelet transform allows the model to simultaneously capture both long-term trends and instantaneous details of the signal, providing TCN with richer input features. Second, to solve the core problem of traditional classification models struggling with unknown and dynamically changing emitter counts, they designed a clever masking generation module at the output end of the TCN. This module can dynamically predict an active emitter “mask” for the current scenario based on the input sequence characteristics, enabling the model to adaptively output a variable number of deinterleaving results, rather than being restricted to a fixed number of classes. This work demonstrates that by introducing prior knowledge from signal processing (wavelet transform) to optimize feature representation and combining it with flexible output mechanisms to overcome task limitations, it is an effective way to improve the performance of non-recurrent convolutional architectures.

**Figure 10:**
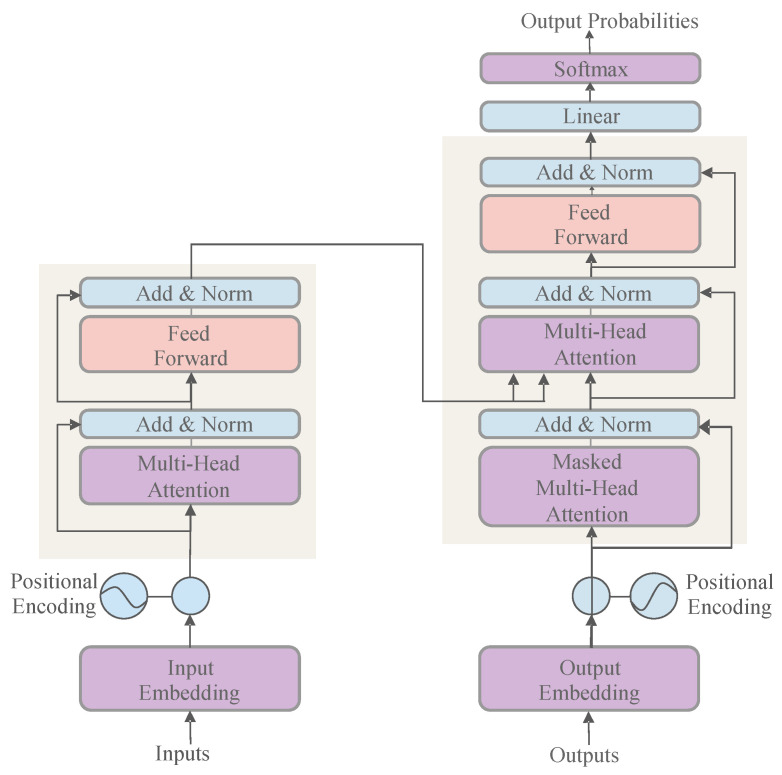
Complete Structure of the Transformer [81].

The complete structure of the Transformer architecture is illustrated in Figure 10. Therefore, the core advantage of the Transformer architecture lies in its powerful inter-pulse relationship modeling capability. Its self-attention mechanism completely liberates it from the sequential processing constraints of RNNs, allowing direct information interaction between any two pulses in the sequence. This enables efficient and parallel capture of non-contiguous association patterns that extend far beyond conventional PRIs (e.g., complex grouped PRIs, PRI revisit periods). In this process, intra-pulse features (e.g., RF, PW, PA) are not ignored but serve as critical components in constructing the multi-dimensional representation (Embedding) of each pulse. The model uses the similarity of these intra-pulse features as a basis for calculating attention weights to judge the correlation strength between pulses. In other words, the Transformer’s architectural design makes it a naturally excellent inter-pulse relationship reasoner, treating intra-pulse features as static “attributes” for judging this relationship. This differs from models like CNNs discussed later, which may focus more on treating intra-pulse features themselves as structured “objects” to be analyzed (e.g., analyzing intra-pulse modulation via time-frequency spectrograms).

### 4.3. Visual Pattern Recognition Based on Convolutional Neural Networks (CNNs)

The core advantage of Convolutional Neural Networks (CNNs) lies in their ability to effectively extract spatial hierarchical features from images. To utilize CNNs, the one-dimensional radar pulse sequence must first be converted into a format that CNNs can process, primarily through two approaches: imaging and point clouding. Imaging is the most common method. It maps two key parameters of pulses (e.g., TOA and RF) onto the x and y axes of a 2D plane, forming a scatter plot [22]. Pulses from the same emitter, due to their inherent regularity and similarity, tend to cluster together on the image, forming “point clusters” or “strips” with specific patterns. By rasterizing this scatter plot, a 2D image suitable for CNN input can be obtained. The advantage of this method is its intuitiveness and its ability to transform complex signal relationships into spatial patterns easily understood by CNNs. However, imaging also has drawbacks: the quantization process may lose precision; sparse pulses result in a large number of meaningless background pixels; and the output is sensitive to image resolution. To overcome these issues, point clouding offers another more direct approach [87,88]. A point cloud is a set of points in a multi-dimensional space, where each pulse can be directly defined as a point by its multi-dimensional parameters (e.g., TOA, RF, DOA). This representation is raw and unstructured, directly preserving the precise parameter values of each pulse and avoiding information loss.

#### 4.3.1. Deinterleaving Models Based on Image Segmentation: From U-Net to Mask R-CNN

**Figure 11:**
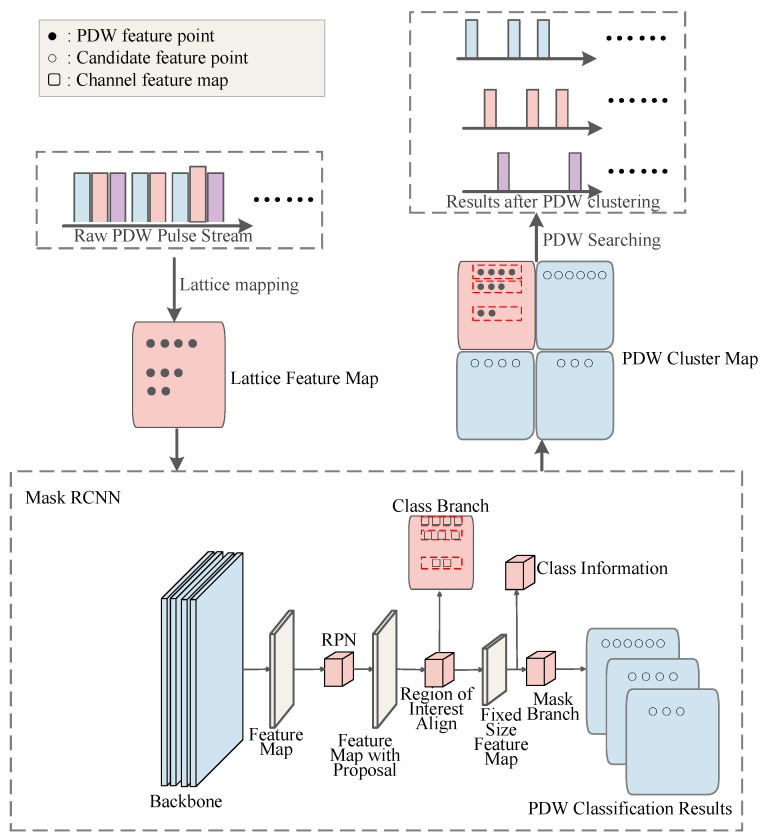
Framework Diagram of Mask R-CNN Network.

Based on the imaging representation, different computer vision task paradigms can be employed for deinterleaving [22,89,90]. The most advanced methods model this as a segmentation task, achieving end-to-end deinterleaving. For the imaging representation, U-Net (a classic semantic segmentation network) has been pioneered for radar signal deinterleaving [22,90]. Using the U-Net model, Kang et al. achieved a segmentation accuracy of 98.53% on the validation set in five-target scenarios [89]. For imaging representation, U-Net, a classic semantic segmentation network, was pioneered for radar signal deinterleaving. U-Net’s encoder-decoder structure and skip connections enable it to precisely segment regions representing different emitters in the image. Its symmetric structure allows the model to utilize both deep, abstract semantic information (from the bottom of the encoder) and shallow, high-resolution detailed information (from the top of the encoder) when performing pixel-level predictions, which is crucial for accurately outlining the boundaries of pulse clusters. However, semantic segmentation can only differentiate classes but not instances of the same class. To address this limitation, the overall framework of the instance segmentation-based Mask R-CNN model is illustrated in Figure 11.

To address this, Chen et al. further modeled the deinterleaving problem as an instance segmentation task, using Mask R-CNN to directly detect and segment pulse clusters of each emitter on the pulse scatter plot [24,91]. In the study by Chen et al. [18], an instance segmentation network based on Mask R-CNN was proposed, offering an end-to-end solution that addresses the limitations of pre-set thresholds and algorithmic structural constraints. Simulation analysis indicates that for sample data of six radar signal types, the algorithm achieves a deinterleaving success rate exceeding 90% under a pulse loss rate of less than 40% or a spurious pulse rate of less than 30%. The Mask R-CNN network framework is shown in the following figure [18]. Mask R-CNN is a two-stage framework: the first stage uses a Region Proposal Network (RPN) to identify candidate regions (bounding boxes) that might contain targets; the second stage classifies each candidate region and generates a pixel-level mask for it. This method can naturally handle unknown emitter counts (the number of detected targets equals the number of emitters) and exhibits good separation capability for signals with overlapping parameters but slight spatial separation. It is one of the most powerful and flexible paradigms for image-based deinterleaving methods.

#### 4.3.2. Direct Deinterleaving Models Based on Point Cloud Processing

For point cloud representation, point cloud segmentation networks such as PointNet and its subsequent improvements (e.g., PointNet++) can be utilized [87,88]. These networks are designed to directly process unordered point sets, overcoming the limitation of traditional CNNs requiring regular grid inputs. The core idea of PointNet is to design a function that is invariant to the order of input points. It independently extracts high-dimensional features for each point using shared multi-layer perceptrons (MLPs) and then aggregates features from all points using a symmetric function (e.g., max pooling) to obtain a global feature vector describing the entire point cloud [92]. For segmentation, PointNet concatenates this global feature with the local features of each point and then uses several MLP layers to predict the class label for each point. PointNet++ recursively applies PointNet in a hierarchical manner, introducing the capability to capture local neighborhood structures, thus learning more refined geometric features. In the deinterleaving task, this is equivalent to directly segmenting pulse points in three-dimensional or higher-dimensional parameter spaces, with each segmented sub-point cloud corresponding to an emitter. This method avoids the information loss caused by imaging and theoretically better captures the geometric structure of data in high-dimensional spaces, representing an important development direction for visual pattern recognition methods. However, challenges include processing efficiency for large-scale point clouds and effectively integrating the temporal dimension (TOA) into point cloud networks that primarily focus on spatial structures. In the experiments conducted by Chen et al. [88], for seven radar signal types, under a Signal-to-Noise Ratio (SNR) of 10 dB and a pulse loss rate of 20%, PointNet++ achieved a deinterleaving accuracy of 94.81%. The visual paradigm provides a unique perspective, unifying the spatial distribution patterns formed by inter-pulse parameters (point locations) with the individual attributes reflected by intra-pulse features (point color, intensity, etc.) on “one image”. It leverages the powerful spatial feature extraction capabilities of CNNs for integrated processing.

### 4.4. Complex Relationship Modeling Based on Graph Neural Networks (GNNs)

#### 4.4.1. Construction Strategies for Pulse Relationship Graphs

GNNs provide a more powerful and flexible framework for modeling arbitrary and complex relationship structures between pulses. The corresponding network schematic diagram [93] is shown as follows. In this paradigm, each pulse is treated as a node (Node) in a graph, and some relationship between pulses is defined as an edge (Edge) connecting the nodes. The radar signal deinterleaving problem is thus elegantly transformed into a graph node clustering (Node Clustering) or community detection (Community Detection) problem [94,95]. The first and most crucial step in GNN methods is to construct the pulse relationship graph. The quality of graph construction directly determines the upper limit of GNN performance.

**Figure 12:**
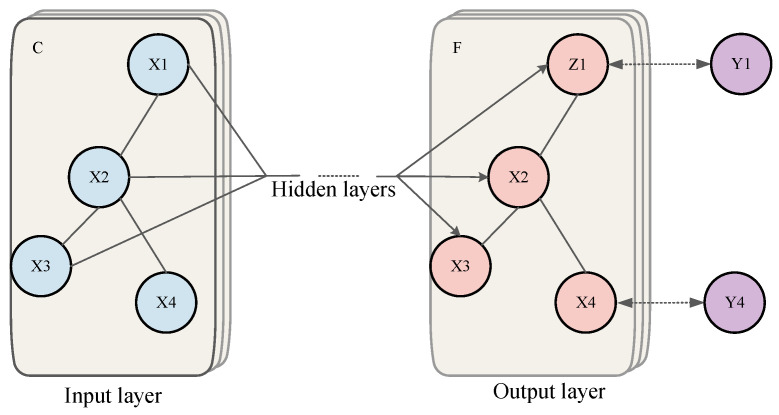
Schematic depiction of multi-layer Graph Convolutional Network (GCN) for semi-supervised learning with C input channels and F feature maps in the output layer. The graph structure (edges shown as black lines) is shared over layers, labels are denoted by $Y_{i}$ [93].

Common construction methods include: (1) k-Nearest Neighbor Graph (k-NN Graph): Each pulse node is connected only to its k nearest neighbors in the multi-dimensional parameter space. This method is simple and can capture local structures, but the choice of k is critical and may overlook pulses that are distant but have strong correlations. (2) Fully-Connected Graph: All pulse nodes are connected to each other, with edge weights determined by their similarity (e.g., Gaussian similarity function). This method preserves all possible relationships but introduces many noisy edges and has huge computational costs. (3) Hybrid Graph: Combines multiple relationships, for example, considering both parameter space proximity and temporal PRI relationships. If the TOA difference between two pulses is close to a candidate PRI value, an edge is added between them. This approach explicitly encodes domain knowledge into the graph structure. How to adaptively and robustly construct a high-quality pulse relationship graph is one of the core challenges of applying GNNs to deinterleaving. Once the pulse relationship graph is constructed, Graph Neural Networks (GNNs), such as Graph Convolutional Networks (GCNs), can be employed to perform feature propagation and node-level inference, as illustrated in Figure 12.

#### 4.4.2. Node Representation Learning Based on Graph Convolutional Networks (GCNs)

After constructing the pulse relationship graph, the core task of GNNs is to learn the representation (Embedding) of each node. GCNs are one of the most representative models in GNNs [23,96]. They aggregate neighbor information in a fixed manner determined by the graph structure. In each layer of a GCN, the new feature vector of a node is obtained by averaging its previous layer’s feature vector and the previous layer’s feature vectors of all its neighbors, followed by a non-linear activation function. The weights for this averaging are typically obtained after normalizing the graph’s adjacency matrix and degree matrix. After multiple layers of such information propagation, the representation vector of each node eventually incorporates information from its k-hop neighborhood graph structure. Theoretically, pulse nodes belonging to the same radar, due to their tight connections on the graph, will have their representation vectors become increasingly similar in the feature space through GCN information propagation. Finally, a simple clustering algorithm (e.g., K-Means) or a classification layer applied on top of the high-quality node representations learned by GCN can complete the deinterleaving. The method based on GCN proposed by Li et al. [96] effectively combines the powerful relationship modeling ability of graph networks with the advantage of semi-supervised learning’s low dependence on label data. Under the conditions of five different radars and a 10% pulse loss rate, the sorting accuracy exceeds 90%. To solve the problem of Radar Signal Deinterleaving in complex electromagnetic environments, especially in scenarios with multiple targets and interference, an efficient and accurate solution is provided. Lang et al. proposed a deinterleaving method based on residual graph convolutional networks [23], while Li et al. combined GCNs with semi-supervised learning, using a few labeled pulses to guide the clustering of the entire graph [96].

#### 4.4.3. Adaptive Information Aggregation Based on Graph Attention Networks (GATs)

A potential limitation of standard GCNs is that they aggregate neighbor information with fixed weights for all neighbors. However, in reality, different neighbors have different significance to the central node. GATs introduce attention mechanisms to address this problem, allowing nodes to assign different, dynamically calculated attention weights to different neighbors when aggregating neighbor information [97]. Specifically, for a central node, GAT calculates an attention coefficient between it and each of its neighbors. This coefficient is typically learned by a small neural network and measures the importance of that neighbor to the central node. These coefficients are then normalized via Softmax to obtain the final attention weights. The new representation of the central node is a weighted sum of its neighbors’ representations, with weights being the attention weights just calculated. This adaptive weighted aggregation allows GATs to have stronger expressive power and generalization ability than GCNs because it enables the model to autonomously learn which edges are more important in the graph. To further enhance the model’s expressive power, GATs typically also employ Multi-Head Attention mechanisms. Huang et al. proposed a hybrid model combining GCN and GAT [97], and Qi et al. [98], based on the multi-head residual GAT, maintained a sorting accuracy rate of 91.66% for four different radar signals at a marking rate of 10%, demonstrating the model’s strong capability. The fundamental advantage of GNN methods is that they explicitly model inter-pulse relationships as graph structures, allowing flexible capture of various complex dependencies and providing a very promising avenue for solving deinterleaving problems in highly complex overlapping scenarios. The power of GNNs lies in their ability to flexibly define inter-pulse “relationships” based on domain knowledge, allowing for the definition of edges based on inter-pulse parameter similarity, as well as edges based on intra-pulse feature correlation, thereby enabling efficient reasoning and aggregation of both types of information within a unified graph structure.

### 4.5. Summary and Comparative Analysis of Deep Learning Models

The preceding sections have systematically detailed the application of mainstream deep learning architectures to the radar signal deinterleaving task, each approaching the problem from a distinct modeling perspective. The performance metrics summarized in Table 5 reveal a clear trend: the evolution from simple sequential processing to more sophisticated visual and relational paradigms has endowed deinterleaving systems with unprecedented robustness and accuracy, particularly under the challenging conditions of modern complex electromagnetic environments. This section provides a comparative analysis to synthesize these findings and highlight the core trade-offs.

The sequential modeling paradigm, including RNNs, Transformers, and TCNs, directly addresses the temporal nature of pulse streams. While foundational models like LSTM excel in capturing basic PRI patterns, their performance is constrained by sequential computation and challenges with long-range dependencies. The introduction of the Transformer architecture marks a significant leap, leveraging global self-attention to overcome these limitations. As evidenced by the results, a Transformer can maintain high accuracy (89%) even with an extreme pulse loss rate of 80%, demonstrating its superior robustness in high-density, high-overlap scenarios. TCNs, as seen in [82], offer a compelling alternative, achieving high performance with greater computational efficiency by using dilated convolutions, making them highly suitable for parallel processing of complex time series.

The visual and spatial modeling paradigm transforms the abstract signal problem into a concrete pattern recognition task. This approach has proven highly effective, with methods like Mask R-CNN achieving over 90% accuracy even with significant pulse loss or spurious pulses. The key innovation here is the shift from semantic to instance segmentation, which elegantly solves the critical problem of handling an unknown number of emitters. Similarly, the direct processing of pulse data as point clouds avoids the information loss associated with imaging. The substantial performance increase from PointNet to PointNet++ underscores the critical importance of capturing local neighborhood structures in the high-dimensional parameter space for fine-grained clustering.

Finally, the relational modeling paradigm, represented by GNNs, offers the most flexible framework by explicitly constructing a graph of inter-pulse relationships. This allows for the capture of complex, non-sequential correlations that might be missed by other architectures. The performance of GNNs is highly contingent on the quality of the graph construction. The evolution from GCNs with fixed aggregation weights to GATs with adaptive, attention-based weighting demonstrates a clear path toward enhancing performance by enabling the model to dynamically focus on the most salient relationships within the data.

In summary, this comparative analysis reveals that no single model is universally optimal. The choice of architecture is intrinsically linked to the specific challenges of the operational environment and the desired performance trade-offs. Sequential models are ideal for PRI-dominant scenarios; visual models excel in handling severe parameter overlap when spatial or feature-space separability exists; and relational models provide the ultimate flexibility for uncovering complex, arbitrary correlations. This understanding of model-specific strengths and weaknesses lays the groundwork for the advanced and emerging paradigms to be discussed in the subsequent chapters.

#### Impact of Input Data Representation

A critical yet often overlooked factor in deep learning performance is the input encoding strategy, which dictates the model’s generalization capability.

- Sequential Encoding (RNNs/Transformers/TCNs): These models consume raw PDW streams or TOA-difference sequences. This representation preserves the highest precision of pulse parameters. However, it is highly sensitive to the *order* of arrival. If a pulse is missing or a spurious pulse is inserted, the relative time differences change globally, potentially disrupting the entire sequence context.
- Visual Encoding (CNNs): Methods like Mask R-CNN convert pulse streams into 2D time-frequency images or TOA-DOA scatter plots. The advantage is that small parameter jitters only result in slight pixel displacements, offering natural robustness. The downside is the loss of precision due to pixel quantization and the inability to separate overlapping pulses that fall into the same pixel grid.
- Graph Encoding (GNNs): Here, each pulse is a node with high-dimensional attributes (RF, PW, DOA), and edges represent potential associations. This is the most flexible representation, as it is invariant to input order and does not suffer from quantization loss. It effectively shifts the burden from “feature extraction” to “relationship reasoning,” making it the most promising direction for complex, agile signal environments.

Data Availability and Training Scales: It is important to note that the deep learning methods reviewed in this section primarily relied on self-generated simulated datasets for training and evaluation, as standardized public benchmarks were historically unavailable. The size of training sets in these studies typically ranges from $10^{3}$ to $10^{7}$ pulses, generated based on varying simulation parameters (e.g., different modulation types and noise levels). Consequently, direct performance comparison across different papers is challenging. To address this issue, the standardized datasets introduced in Section 5 (such as the EBDSC dataset) have recently been released to provide a unified benchmark for future research.

## 5. RSS Datasets

The application of deep learning algorithms is highly dependent on large-scale, measurable, standardized, and precise training samples. Therefore, obtaining labeled training data is considered the primary prerequisite for advancing deep learning methods in the field of Radar Signal Deinterleaving (RSS). Although deep learning methods have seen many successful research and application efforts in radar, most studies still use simulated datasets. Overall, there are relatively few publicly released RSS datasets. This section focuses on introducing one high-quality dataset currently available.

### 5.1. Dataset Descriptions

#### 5.1.1. The 2nd Electromagnetic Big Data Super Contest (EBDSC) Dataset

The Datacastle dataset [99] was provided by the 2nd EBDSC Dataset. The competition was jointly organized by the 29th Research Institute of China Electronics Technology Group Corporation, Xi’an University of Electronic and Science Technology, and other institutions. This dataset includes twelve types of radar signals, as shown in Table 6. The dataset also provides three scenarios as test sets to simulate interleaved radar pulse streams in real-world environments. For the training data: Each signal type from each radar is set to transmit for 10 s. The dataset contains samples of 12 signal types, with each type stored in a separate .txt file, totaling 12 .txt files. Each training file consists of a series of time-series data points. Each data point is primarily described by six feature parameters, as shown in the figure below, from left to right: timestamp TOA, Feature A, Feature B, Feature C, Feature D, and Label ID. Each file contains only noise-free, pure, single-class signals of that type (no overlap, loss, errors, or interference).

To rigorously evaluate deinterleaving performance under varying overlapping densities, the validation dataset is structured into three distinct difficulty levels: Simple, General, and Complex. The simulation environment is configured with the following specific characteristics:

- Signal Environment: The scenarios simulate airborne targets with independent on/off switching mechanisms, resulting in random signal overlapping across time, space, and frequency domains. While the signal templates are consistent with the training set, the test data introduces realistic perturbations, including Gaussian noise, parameter measurement errors, and a small fraction of outlier parameters.
- Dynamic Constraints: Target Direction of Arrival (DOA) is modeled as either fixed or continuously changing to mimic realistic flight trajectories.
- Signal Quality: The Signal-to-Noise Ratio (SNR) is maintained at a challenging level of 5 dB, testing the algorithms’ robustness against noise.

The specific composition of the three test datasets is detailed as follows:

- Test Set I (Simple): Comprises interleaved pulse streams from 4 distinct emitter types (IDs: 0, 2, 5, 7). The total data volume is 999,038 pulses.
- Test Set II (General): Involves a higher density mixture of 8 emitter types (IDs: 0, 1, 2, 4, 5, 6, 7, 8), increasing the deinterleaving complexity. The total data volume is 1,762,620 pulses.
- Test Set III (Complex): Represents the most challenging scenario with all 12 emitter types active simultaneously, creating severe pulse overlap. The total data volume reaches 2,579,053 pulses [100].

#### 5.1.2. Proposal for Standardized Benchmark Protocols

To address the reproducibility crisis in this field, we propose that future research utilizing the datasets mentioned above should adhere to a standardized evaluation protocol. We recommend that algorithm performance be reported not just as a single accuracy metric, but as a function of environmental complexity. Specifically, authors should report:

- Robustness Curves: Performance under varying pulse loss rates (e.g., 10%, 30%, 50%) and spurious pulse ratios.
- SNR Sensitivity Evaluation: Performance evaluated across a wide range of signal-to-noise ratios, from −20 dB to 20 dB, with a step size of 2 dB. Results should be reported at each SNR level to characterize degradation trends and low-SNR robustness.
- Metric Standardization: Use the Adjusted Rand Index (ARI) as the primary metric for clustering quality, as it accounts for chance grouping, rather than simple classification accuracy which can be misleading in imbalanced scenarios.

Adopting these protocols will allow for a fair and rigorous comparison between the diverse architectures discussed in Section 4.

### 5.2. Summary of RSS Datasets

Research on Radar Signal Deinterleaving (RSS) methods relies heavily on datasets. However, due to the specific nature of the RSS task, besides the datasets mentioned above, there are no typical publicly available actual PDW datasets for deep learning research. Most RSS datasets come from electromagnetic simulations and field acquisitions conducted by different research teams. Because channels for acquiring radar target features are scarce and measurement processes are extremely challenging, migrating existing models based on simulated data to actual data is of significant research value. Datasets are the cornerstone of deep learning method development. Currently, public, standardized real radar signal datasets are relatively scarce, which somewhat constrains the depth of research and the validation of algorithm universality. More high-quality datasets are needed in the future.

## 6. Advanced and Emerging Radar Signal Deinterleaving Paradigms

With the deepening of research, the field of radar signal deinterleaving is no longer limited to improving single models but has seen the emergence of numerous new paradigms that draw upon advanced ideas from other areas of machine learning. These new paradigms aim to address deeper challenges in the deinterleaving task, such as adaptability to new emitters, utilization of prior knowledge, fusion of multi-source information, and model interpretability.

### 6.1. Data-Efficient Learning Paradigms

The great success of deep learning models is typically built upon massive amounts of labeled data. However, in the field of radar signal deinterleaving, obtaining large-scale, high-quality labels is extremely difficult. Therefore, developing data-efficient learning paradigms that reduce reliance on labeled data is key to advancing deinterleaving technology towards practical applications.

#### 6.1.1. Unsupervised Data Representation Learning Based on Self-Supervision

Self-Supervised Learning (SSL) provides a powerful solution to the problem of “data hunger”. The core idea of SSL is to design a “pretext task” from unlabeled data itself, without relying on manual annotations, and by solving this pretext task, force the model to learn useful and general data representations (Representations) for downstream tasks. Contrastive Learning is currently one of the most mainstream and successful paradigms in SSL. Its basic idea is “birds of a feather flock together”: through data augmentation, one or more “positive examples” (i.e., samples that have undergone different transformations but are semantically similar) are created for each sample (termed “anchor”). All other samples in the dataset are then considered “negative examples”. An encoder is then trained with the objective of pulling the representations of the anchor and its positive examples as close as possible in the feature space, while pushing them as far away as possible from all negative examples. For radar pulse sequences, data augmentation methods can include: random discarding of some pulses, applying minor jitter to TOA, and adding noise to parameter values. Through this contrastive learning approach, the model can learn an excellent feature representation that is invariant to the intrinsic signal patterns and various perturbations, even without a single manual label.

A notable application of this paradigm to radar deinterleaving was recently demonstrated by Gunn et al. [101] through the use of Transformer-based deep metric learning. In their work, a Transformer architecture, adept at capturing the complex temporal dependencies within pulse streams, serves as the deep feature extractor. This network is trained using a metric learning objective, a form of contrastive learning, to map the input pulse sequences into a high-dimensional embedding space. The training objective ensures that in this learned space, sequences from the same emitter form tight, compact clusters, while sequences from different emitters are pushed far apart. Consequently, the complex, non-linear deinterleaving problem is transformed into a much simpler one of identifying geometric separation in the learned latent space. Once the model is trained, even a simple clustering algorithm like K-Means applied in this embedding space can achieve highly accurate deinterleaving results. Yang et al. proposed a deep contrastive clustering method for signal deinterleaving, which deeply integrates contrastive learning with the clustering process. In a unified framework, feature learning and cluster assignment are optimized end-to-end, achieving significantly better deinterleaving performance than traditional clustering methods in an unsupervised manner. Dai et al. also explored unsupervised deinterleaving methods that synergistically optimize feature fusion and data augmentation, embodying the ideas of self-supervised learning [102]. These methods greatly reduce reliance on expensive labeled data and enhance the applicability of models in real, complex, unlabeled environments.

#### 6.1.2. Fast Adaptation and Few-Shot Deinterleaving Based on Meta-Learning

Meta-Learning, or “Learning to Learn,” aims to enable models to learn general prior knowledge or learning strategies from multiple related tasks, so that when encountering new, unseen tasks, they can use this knowledge for rapid adaptation, typically requiring only a few new samples. This is of critical importance for responding to constantly emerging new radar systems in the rapidly changing battlefield environment. By training a meta-learning model on a large number of simulated, diverse deinterleaving tasks, it can grasp the essential principles of the deinterleaving problem. When this “experienced” model is deployed in a real, entirely new electromagnetic environment, even when encountering a novel radar system, it can quickly adjust itself by observing only a few (Few-Shot) pulses from that radar, thereby forming an effective deinterleaving capability for that new radar. Qi et al. explored meta-learning-based radar emitter signal deinterleaving methods by constructing a series of simulated deinterleaving tasks to train meta-learners, enabling them to adapt quickly to new scenarios [103]. This provides an effective approach for solving few-shot deinterleaving problems, i.e., deinterleaving rare or novel radars with very few samples.

#### 6.1.3. Effective Utilization of Limited Labels with Semi-Supervised and Active Learning

Between fully unsupervised (no labels) and fully supervised (large amounts of labels), there exists a broad intermediate ground: Semi-Supervised Learning. Semi-supervised learning aims to learn using both a large amount of unlabeled data and a small amount of labeled data simultaneously. Its underlying assumption is that labeled and unlabeled data share some underlying structure. For example, if two points are very close in the feature space, their labels should also be very similar. Based on this, knowledge learned from labeled data can be smoothly extended to unlabeled data through graph propagation, consistency regularization, and other methods. Li et al. combined GCNs with semi-supervised learning, using a few labeled pulses as “seeds” to guide the clustering of the entire graph through information propagation on the graph, significantly improving deinterleaving performance [96]. Active Learning is another effective strategy for utilizing a small amount of labels. It attempts to let the model “actively” select the samples that are “most uncertain” or “most informative” and present them to experts for annotation. Through this approach, the maximum performance improvement for the model can be achieved with minimal annotation cost. In radar signal deinterleaving, this means the system can automatically present the most ambiguous and difficult-to-deinterleave pulse segments to the operator, thus achieving efficient human-in-the-loop processing. Both these strategies provide feasible paths for maximizing the utility of deep learning models under extremely limited label resources in real-world conditions.

### 6.2. System-Level Fusion and Collaborative Paradigms

To overcome the performance bottlenecks of single-station systems, integrating processing with multi-source information and synergistically optimizing deinterleaving with related tasks are inevitable choices for enhancing overall system performance.

#### 6.2.1. Multi-Station Data Fusion and Collaborative Deinterleaving

The core advantage of multi-station collaborative deinterleaving lies in utilizing the differences in observations of the same emitter from geographically dispersed stations to provide additional deinterleaving dimensions unobtainable by single-station systems [41,42,104]. Among these, Time Difference of Arrival (TDOA) and Difference of DOA are the two most critical pieces of information. Due to varying distances from each station to the emitter, the TOA of the same pulse arriving at different stations will differ; this TDOA information is key for passive emitter localization and can also serve as a strong feature for distinguishing emitters at different spatial locations. Multi-station information fusion can be performed at different levels, including data-level, feature-level, and decision-level, each with its own advantages and disadvantages. With the advancement of deep learning, researchers are beginning to use neural networks to automatically learn how to effectively fuse feature information from different stations. For example, Li et al. researched multi-station collaborative radar signal deinterleaving methods based on deep information fusion [104]. Mu and Qu cleverly utilized TDOA and DOA information to propose a 3D DBSCAN algorithm that directly incorporates spatial location information into the clustering process, achieving tight integration of deinterleaving and localization [41]. Multi-station collaboration not only provides richer features but also overcomes observation blind spots and interference of individual stations through spatial diversity effects, representing an inevitable choice for future high-performance electronic reconnaissance systems.

#### 6.2.2. Joint Optimization of Deinterleaving and Localization

In modern electronic reconnaissance systems, signal deinterleaving is not an isolated endpoint task but a key intermediate link in the entire intelligence generation chain. Traditional “serial” processing pipelines suffer from error accumulation and information isolation. To overcome these issues, jointly optimizing (Joint Optimization) deinterleaving and localization within a unified framework for end-to-end processing is a crucial future trend. This joint processing paradigm enables synergistic and complementary information exchange between tasks, thereby enhancing overall system performance. For example, Zhao et al. proposed a framework that jointly performs pulse association, deinterleaving, and localization, solving these three sub-problems simultaneously within a unified model, achieving mutual enhancement of information [105]. Preliminary localization results can provide strong spatial priors for deinterleaving (e.g., predicting the TOA and DOA of pulses from that location), helping to resolve parameter overlap issues. Conversely, accurate deinterleaving results (grouping pulses belonging to the same emitter) can significantly improve the accuracy and robustness of TDOA localization. This joint optimization idea breaks down the barriers of traditional modular processing, allowing the system to configure resources and make decisions from a global optimization perspective, representing a key step towards intelligent, integrated electronic reconnaissance.

#### 6.2.3. End-to-End Processing of Deinterleaving and Identification

Similar to localization, emitter identification is a direct downstream task of deinterleaving, aiming to determine which specific type of radar each deinterleaved pulse sequence belongs to (e.g., airborne fire control radar, ground search radar). Traditionally, this is also a serial process. However, end-to-end processing frameworks attempt to perform deinterleaving and identification within a single model. Scholl et al. focused on achieving end-to-end multi-label classification, integrating deinterleaving and identification (even simultaneously identifying multiple emitters in a scene) into a single model [106,107]. The model, while learning deinterleaving, also learns to identify the type of each emitter, and these two tasks can share the underlying feature extraction network, mutually promoting each other. For instance, learning for the identification task can drive the feature extraction network to focus on intra-pulse “fingerprint” features that are more critical for distinguishing radar models, and these features may, in turn, contribute to more refined deinterleaving. This multi-task learning framework not only improves processing efficiency but also enhances model generalization ability through task-based regularization effects. The ultimate goal is for the system to receive a mixed pulse stream and directly output high-level intelligence such as “At azimuth X, range Y, there is an Type A radar operating in Mode Z,” rather than a series of intermediate, isolated parameters.

### 6.3. Emerging Explorations Towards Cognitive Intelligence

Beyond innovations in learning paradigms and system architectures, some more cutting-edge and exploratory concepts are beginning to emerge in radar signal deinterleaving, signaling an evolution towards higher-level cognitive intelligence.

#### 6.3.1. Challenges in Model Interpretability

Explainable AI (XAI) is crucial for the practical deployment of radar signal deinterleaving models [106]. In high-risk fields such as military applications, decision-makers not only need to know what the deinterleaving result is but also need to understand why the model made that judgment. Developing interpretable techniques, such as visualizing attention weights to analyze which pulses the model primarily “focused on” during decision-making, or generating counterfactual explanations to illustrate what changes are needed in the input to alter the deinterleaving result, helps build model trust and transparency. This is fundamental for establishing effective human-machine collaborative decision-making systems. It not only concerns user trust in AI systems but also relates to the prediction and control of model behavior in complex adversarial environments.

#### 6.3.2. Enabling Low-Power and Edge Computation

Spiking Neural Networks (SNNs), as a third generation of neural networks, offer a computational paradigm closer to the working mechanism of the biological brain. Their event-driven and sparse computation characteristics give SNNs huge theoretical advantages in energy efficiency, making them particularly suitable for real-time implementation on power-constrained edge devices (e.g., drones, satellites). Luo and Liu applied interpretable SNNs to radar pulse stream deinterleaving, exploring their feasibility and advantages in this task [108]. The event-driven nature of SNNs naturally aligns with the discrete arrival characteristics of radar pulses, promising important roles in future edge computing and real-time processing systems, and offering new ideas for building more efficient and bio-inspired deinterleaving models.

#### 6.3.3. Large Language Models Empowering Semantic Cognition and Reasoning for Radar Signals

In recent years, LLMs, exemplified by the GPT series, have reshaped the artificial intelligence landscape owing to their astonishing emergent abilities—such as zero-shot/few-shot learning, contextual understanding, and complex logical reasoning—acquired through pre-training on massive multimodal data. Integrating the capabilities of these powerful LLMs into the domain of radar signal processing presents a highly imaginative and promising frontier direction.

Emerging research has begun to validate this potential. For example, recent studies have explored using LLMs to identify materials from radar signals [109], advanced cognitive radar through natural language processing [110], and empower multimodal integrated sensing and communication systems [111]. Additionally, high-resolution estimation techniques in coherent distributed radar [112] are being integrated with semantic cognition. These works collectively suggest that LLMs can serve as a central cognitive brain, orchestrating low-level signal processing tasks including deinterleaving.

- Knowledge-Enhanced Intelligent Deinterleaving: Leveraging large models as external knowledge bases to provide prior knowledge for deinterleaving systems, guiding the deinterleaving process, and even enabling “zero-shot” deinterleaving of unknown radars. For instance, a system can receive natural language descriptions from intelligence sources (e.g., “Attention required to locate a Russian new-generation fire control radar operating in the X-band, employing a 5-bit Barker code, with PRI jitter between 200–300 microseconds”). The foundation model can parse this semantic description into specific parameter constraints and feature patterns, thereby guiding or configuring underlying deinterleaving algorithms for targeted “zero-shot” searches, significantly enhancing the efficiency of detecting specific threats.
- Semantic Interpretation and Behavioral Reasoning of Deinterleaved Results: Foundation models can automatically translate tedious parameter sequences (e.g., PRI lists) from deinterleaved results into human-understandable, structured “natural language” tactical reports, enabling large models to comprehend and reason about radar behavioral patterns [113,114].
- Human-Machine Collaborative Cognitive Electronic Warfare: Through natural language interaction, operators can issue vague or complex commands to large model-based deinterleaving systems, inquire about decision bases, and conduct hypothetical reasoning (“What-if” analyses). The system can then understand intentions, execute tasks, and provide interpretable feedback. This constitutes the cornerstone for achieving advanced human-machine collaboration and advancing towards cognitive electronic warfare. Table 7 provides a summary of these advanced and emerging paradigms.

Although research in this area is still in its nascent stages, preliminary yet crucial explorations have been undertaken by Niu Kairui et al. [115]. They proposed a large model-inspired enhanced radar signal deinterleaving method. The core of this approach lies in utilizing the powerful prior knowledge and generative capabilities of large models for high-quality data augmentation of sparse or imbalanced training data, thereby improving the performance of downstream deinterleaving models. Their designed model incorporates multi-scale fusion embeddings and a masking enhancement mechanism. In a complex scenario involving 12 types of radar signals with a signal-to-noise ratio of only 5 dB, it achieved a correct deinterleaving rate of up to 94%. This work powerfully validates that using large models to drive data augmentation as an entry point is an effective strategy for addressing the small-sample problem in the electromagnetic domain, and it also represents the first successful attempt at translating this concept into practice. The deep integration of foundation models with signal processing undoubtedly opens up novel and exciting pathways for achieving higher-level battlefield situation understanding and intelligent decision-making.

## 7. Comprehensive Comparison and Analysis

After detailed discussions in the preceding chapters, we have gained a deep understanding of the various radar signal deinterleaving methods. This chapter aims to provide a horizontal, global comparison and analysis of these methods, offering a comprehensive summary in Table 8 to reveal the intrinsic trade-offs of different technical routes, thereby providing a reference for researchers and engineers in selecting or designing algorithms for practical applications.

From the comprehensive comparison in the table above, we can draw the following key analyses:

- Shift from Deterministic to Data-Driven: Most traditional methods are based on deterministic manual rules or statistical models (e.g., PRI periodicity, parameter distances), with their performance ceiling limited by the effectiveness of these rules. In modern complex environments, these rules often fail. Deep learning methods are data-driven; they do not rely on explicit rules but automatically learn complex, nonlinear mapping relationships from large amounts of data, thus possessing stronger adaptability and higher performance ceilings.
- Evolution of Feature Engineering: Traditional clustering methods rely heavily on feature engineering, i.e., selecting which parameters (DOA, RF, PW, etc.) and how to weight and normalize them. This process requires extensive expert knowledge and is often suboptimal. Deep learning, especially end-to-end models, can achieve automatic feature learning and hierarchical abstraction, transforming raw PDW parameters into advanced feature representations that are more beneficial for deinterleaving tasks, significantly reducing reliance on manual feature engineering.
- Trade-off Between Model Complexity and Performance: Overall, from traditional methods to deep learning methods, and then to emerging paradigms, the complexity of models, demand for computational resources, and data dependency are constantly increasing. A simple SDIF algorithm might be implemented in a few lines of code, whereas a Transformer or GNN-based model requires complex network architectures, large amounts of training data, and powerful hardware support (e.g., GPUs). Therefore, in practical applications, a reasonable trade-off must be made between algorithm performance and cost, based on task requirements, resource limitations, and real-time constraints.
- Blurring Boundaries Between Unsupervised and Supervised Learning: Traditional deinterleaving (clustering) is essentially unsupervised learning. The introduction of deep learning has brought numerous supervised learning methods (e.g., sequence labeling, image segmentation), which generally outperform unsupervised methods but require large amounts of labeled data. To resolve this contradiction, semi-supervised learning (such as [96]), self-supervised learning, and unsupervised learning (e.g., deep contrastive clustering) have emerged. These methods attempt to leverage the powerful capabilities of deep learning while reducing reliance on labels, representing an important direction for future development.
- Diversification of Problem Modeling Perspectives: The rise of deep learning has greatly enriched the modeling perspectives for radar signal deinterleaving problems. Beyond traditional clustering, we can now view it as a sequence-to-sequence problem, an image segmentation problem (Vision-based), or a graph community detection problem (Graph-based). Different perspectives bring different solutions and advantages, providing more tools for solving specific challenges. For instance, for scenarios where PRI features are extremely important, sequence models might be the best choice; whereas for scenarios with severely overlapping parameters but spatial separability, vision-based models might be more advantageous.

## 8. Open Issues and Future Research Directions

Despite the significant progress in radar signal deinterleaving technology, numerous open issues remain to be addressed on the path towards full intelligence and autonomy. Synthesizing the analyses from the preceding sections, we identify the following key open issues and future research directions:

- Generalization to Novel Emitters (Open-Set Recognition): Most current deep learning models are trained and tested on a closed-set, pre-defined radar signal dataset. In real battlefield environments, new radar systems or unknown operating modes not present in the training set may appear at any time. The performance of existing models often degrades sharply when faced with these “out-of-distribution” samples, or they may make erroneous “forced” classifications. Therefore, developing deinterleaving algorithms with Open-Set Recognition capabilities is crucial. This requires models not only to accurately deinterleave known emitter classes but also to effectively detect, separate, and cluster unknown signal classes. Meta-learning, Continual Learning, and anomaly detection techniques will be key to solving this problem.
- Data-Efficient Learning (Few-Shot and Zero-Shot Capabilities): For many rare or novel radar signals, we may only be able to obtain very few or even no samples. How to achieve effective deinterleaving under such “data-starved” conditions is a highly challenging problem. Few-Shot Learning aims to enable models to learn discriminative capabilities for a new class from very few samples. Zero-Shot Learning is even more aggressive, expecting models to recognize signals of a new class solely based on semantic descriptions of that class (e.g., parameter ranges, operating mode descriptions). This may require deep integration of signal processing with natural language processing, knowledge graphs, and other technologies [115].
- Real-time Performance and Edge Computing Deployment: Electronic warfare scenarios impose extremely stringent real-time requirements on signal processing. Many advanced deep learning models (e.g., large Transformers, GNNs), while powerful in performance, have enormous computational demands that make real-time processing difficult on resource-constrained edge devices (e.g., airborne or shipborne ESM systems) [116,117]. Therefore, future research needs to focus on model lightweighting, including model compression, knowledge distillation, quantization, and designing more efficient network architectures. Additionally, exploring the combination of low-power computing paradigms like SNNs with deinterleaving tasks, and utilizing dedicated hardware such as FPGAs and ASICs for acceleration, are important avenues for ensuring real-time performance [118].
- Interpretability, Robustness, and Trustworthy AI: As mentioned earlier, the “black box” nature of deep learning models limits their application in critical decision-making domains. Future deinterleaving models must not only “know what” but also “know why.” Developing interpretable AI techniques, allowing users to understand the model’s decision basis, is crucial for establishing human-machine trust and debugging model failures. At the same time, model robustness needs more attention. Research shows that deep models are susceptible to Adversarial Attacks, where adding imperceptible perturbations to input signals can lead to entirely erroneous judgments by the model. Researching the adversarial robustness of deinterleaving models and developing corresponding defense mechanisms are necessary prerequisites for ensuring their reliable operation in complex electromagnetic adversarial environments.
- End-to-End Cognitive Electronic Warfare Systems: Future radar signal processing will move beyond simple serial concatenations of deinterleaving, identification, and threat assessment modules to become a closed-loop system integrating perception, cognition, decision-making, and action. Radar signal deinterleaving, as a core component of perception, requires deeper integration with backend tasks. Designing an intelligent end-to-end system capable of achieving perception from raw pulses to behavioral intent understanding and then to adversarial decision generation is one of the ultimate goals in electronic warfare. This requires interdisciplinary collaboration, integrating knowledge from signal processing, machine learning, control theory, and game theory, and may require drawing inspiration from the “World Models” concept driven by large models to build dynamic perception and prediction capabilities of the electromagnetic environment.
- LLM-Empowered Semantic Reasoning and Strategy: The advent of Large Language Models (LLMs) opens transformative possibilities. Instead of directly processing pulse data, LLMs can serve as a “cognitive core.” They can function as global knowledge-driven agents, fusing intelligence from heterogeneous sources (including external intelligence databases, historical behavioral patterns, and even natural language instructions) to guide the deinterleaving process. By interpreting deinterleaved results in tactical contexts and generating human-understandable reports, LLMs can create autonomous “cognitive loops,” enabling an unprecedented level of self-learning, self-adaptation, and intelligent decision-making in electronic warfare systems.

## 9. Conclusions

Radar signal deinterleaving stands as a pivotal and perpetually evolving challenge at the heart of electronic reconnaissance and countermeasures. This paper has delivered a panoramic and in-depth review, charting the technological trajectory from its foundational principles to the cognitive frontiers of tomorrow. Our journey began with a critical appraisal of traditional methods—spanning PRI search, multi-dimensional clustering, and hybrid strategies—systematically revealing their inherent limitations in the face of the modern complex electromagnetic environment. The review then charted the subsequent paradigm shift toward deep learning, offering a structured and meticulous analysis of how diverse architectures—from sequential models like RNNs and Transformers to visual frameworks like CNNs and relational models like GNNs—have reshaped the problem space. We further explored nascent paradigms in data-efficient learning and system-level fusion, which collectively sketch the blueprint for the next generation of intelligent signal processing. Our central finding is that the field is undergoing a profound metamorphosis: from rule-based determinism to data-driven intelligence, from monolithic problem-solving to diverse modeling perspectives, and from isolated task processing to holistic system integration. Despite these significant advancements, our analysis confirms that the path to fully autonomous and intelligent systems is paved with formidable challenges, notably in generalization to novel signals (open-set capability), few-shot learning, real-time edge deployment, and achieving trustworthy, interpretable AI. The open issues and future research directions articulated in this paper are not merely an epilogue but are intended to serve as a strategic roadmap to guide subsequent inquiry. Looking ahead, we are confident that the continued symbiosis of artificial intelligence and signal processing will propel radar signal deinterleaving systems beyond their current capabilities, transforming them into truly cognitive agents that can master the complexities of the electromagnetic spectrum and secure a decisive advantage in high-stakes environments.

## Figures and Tables

**Table 1:** Primary Parameters.

Abbreviation	Parameter	Notation	Unit
TOA	time of arrival	*t*	µs
DOA	direction of arrival	θ	degrees
PA	pulse amplitude	*a*	dBm
PW	pulse width	*w*	µs
RF	radio frequency	*f*	GHz

**Table 2:** Simulation scenario example, blending three waveforms.

Pulse	TOA (µs)	PW (µs)	RF (MHz)	Emitter
1	103.5	2.3	9471.0	1
2	132.0	42.1	9310.0	3
3	361.2	1.8	9500.0	1
4	416.0	0.5	9400.0	2
…	…	…	…	…
n	9720.0	2.1	9504.0	1

**Table 3:** Summary of PRI Temporal Feature-Based Methods.

Category	Technique	Principle	Advantages	Disadvantages
Histogram-based	CDIF/SDIF	TOA diff stats, peak detection.	Simple; fair pulse loss robustness.	High complexity; pulse loss sensitive; false peaks.
Transform-domain	PRI Transform/Hough	Time periodicity to domain peaks.	False peak suppression; noise robust.	High computation; fixed/linear PRI only.
Sequential Search	Search/Correlation	PRI-tolerant chain search/correlation.	Intuitive; partial PRI handling.	High complexity; start-sensitive; poor overlap handling.

**Table 4:** Summary of Multi-Dimensional Parameter Space Clustering-Based Methods.

Method	Technique	Core Idea	Predefined K	Noise Robust	Complexity
Partitioning	K-Means	Centroid-based	Y	Low	Low
Density-based	DBSCAN	Density-based	N	High	Medium
Graph-based	Spectral	Graph Partitioning	N	Low	High
Hierarchical	Hierarchical	Hierarchy Building	N	Low	High

**Table 5:** Summary of Mainstream Deep Learning Models in Radar Signal Deinterleaving.

Model	Adv	Disadv	RadarNum	Reference	Condition	Training Pulse Count	Accuracy
LSTM	Sequential	Gradients	2	[77]	Pulse loss < 30%	6000	87.00%+
Bi-LSTM	Contextual	Complex	3	[78]	30% miss, 30% false	8,000,000	72.40%
Attn-Bi-LSTM	Focus	Overhead	3	[78]	30% miss, 30% false	8,000,000	74.84%
Transformer	Parallel	High complexity	4	[83]	80% pulse loss	600	89.00%
Modern TCN	Parallel	Weaker non-seq.	12	[82]	SNR 5 dB	1,687,130	88.68%
U-Net	Semantic	Grid, No instance	5	[89]	–	N/A (Code not public)	98.53%
Mask R-CNN	Instance-aware	Complex pipeline	6	[18]	Pulse loss < 40%	2400	90.00%+
PointNet	Unordered	No local context	4	[87]	–	N/A (Code not public)	81.84%+
PointNet++	Local context	More complex	7	[88]	SNR 10 dB, 20% loss	N/A (Code not public)	94.81%
GCN	Relational	Graph dependent	5	[96]	10% pulse loss	3260	90.00%+
GAT	Adaptive	More complex	4	[98]	10% labeling	3260	91.66%

**Table 6:** Parameter Settings for Each Radar Emitter in the 2nd EBDSC Dataset [82].

Radar	DOA/°	PW/µs	RF/MHz	PRI/µs	PA/dBm	Pulse No.
1	54∼61	8∼9	9591∼9619	37∼147	−102∼0	129,720
2	39∼47	8∼10	9610∼9620	37∼148	−115∼0	120,110
3	38∼45	9∼10	9606∼9612	37∼63	−135∼0	193,320
4	44∼51	8∼10	9636∼9643	37∼121	−103∼0	133,054
5	45∼52	9∼11	9592∼9625	37∼113	−117∼0	137,488
6	43∼51	8∼10	9579∼9596	36∼111	−114∼0	143,848
7	57∼63	9∼11	9557∼9564	37∼98	−109∼0	155,580
8	58∼65	9∼10	9566∼9616	7∼41	−132∼0	222,870
9	57∼64	9∼10	9558∼9613	16∼52	−120∼0	150,702
10	51∼58	9∼10	9575∼9580	24∼44	−112∼0	120,392
11	38∼45	1∼2	9579∼9653	7∼39	−132∼0	157,934
12	49∼55	33∼38	2825∼2841	398∼508	−91∼0	22,112
I	43∼67	1∼8	9725∼9743	0∼118	−128∼0	999,038
II	50∼286	1∼9	9082∼9118	0∼143	−129∼0	1,762,620
III	34∼66	1∼39	2807∼9617	0∼116	−149∼0	2,579,053

**Table 7:** Summary of Advanced and Emerging Radar Signal Deinterleaving Paradigms.

Paradigm Category	Core Idea	Key Problems Addressed	References
Data-Efficient Learning	Self-supervised/Meta-learning; Adaptability	Label scarcity; Novel/rare radar adaptation	[102,103]
System-Level Fusion	Multi-station/Task Fusion	System bottlenecks; Error accumulation	[41,104,105,107]
Emerging Cognitive Intelligence	XAI; SNNs; Foundation Models	Interpretability; Low-power; Cognition	[106,108,113,115]

**Table 8:** Summary of Radar Signal Deinterleaving Methods with Inference Speed.

Category	Method	Principle	Advantages	Disadvantages	Scenario	Inference Speed	References
Traditional	PRI Hist. (CDIF/SDIF)	Histogram Peak	Simple/Fast	Fixed PRI only	Stable PRI	Fast	[8,9,40]
	PRI Transform (Hough)	Transform Domain	Noise Robustness	High computation/Parameter Sensitive	Medium Density	Medium	[40,56,57]
	K-Means	Distance Partition	Fast convergence.	Needs K/Non-Convex Fail	Known K	Fast	[11]
	DBSCAN	Density Reachability	Auto K/Irregular Shape	Param Sensitive/Uneven Density	Unknown K	Medium	[10,54,65]
Deep Learning	RNN/Transformer	Temporal Dependency	Captures PRI Patterns	Training Data Heavy	Complex PRI	Medium-Slow	[77,83,85]
	CNN/ PointNet	Sequence to Image/Point Cloud	Overlap Robust/Unknown K	Compute Cost	High Density Overlap	Slow	[22,87]
	GNNs	Inter-pulse Relationship Graph	Flexible Structure Modeling	Graph Cost /Interpretability Poor	Complex Relations	Slow	[23,96,97]
Emerging	Data-Efficient (SSL/Meta)	Reduced Label Reliance	Low Label Cost/Fast Adapt	High Algorithmic Complexity	Scarce Labeled Data	Medium	[102,103]
	System Fusion (Multi-Station/Task)	Multi-Source/Task Optimize.	Improves performance/robustness	High Sync/Design Needs	High-Threat ELINT	Medium-Slow	[41,104,105]

Inference Speed: qualitative estimate (Fast/Medium/Slow/Medium-Slow).

## Data Availability

The data are available in the article.
